# Visibility and attractiveness of *Fritillaria* (Liliaceae) flowers to potential pollinators

**DOI:** 10.1038/s41598-021-90140-7

**Published:** 2021-05-26

**Authors:** Katarzyna Roguz, Laurence Hill, Sebastian Koethe, Klaus Lunau, Agata Roguz, Marcin Zych

**Affiliations:** 1 Botanic Garden, Faculty of Biology, University of Warsaw, Warsaw, Poland; 2 Petersham Lodge, Richmond, UK; 3 Institute of Sensory Ecology, Faculty of Biology, Heinrich-Heine-Universität Düsseldorf, Düsseldorf, Germany; 4 National Information Processing Institute, Al. Niepodległości 188 B, 00-608 Warszawa, Poland

**Keywords:** Ecosystem services, Evolutionary ecology

## Abstract

Visual floral characters play an important role in shaping plant-pollinator interactions. The genus *Fritillaria* L. (Liliaceae), comprising approximately 140 species, is described as displaying a remarkable variety of flower colours and sizes. Despite this variation in visual floral traits of fritillaries, little is known about the potential role of these features in shaping plant-pollinator interactions. Here, we seek to clarify the role of visual attraction in species offering a robust food reward for pollinators early in the spring, which is the case for *Fritillaria*. We also searched for potential tendencies in the evolution of floral traits crucial for plant-pollinator communication. The generality of species with green and purple flowers may indicate an influence of environmental factors other than pollinators. The flowers of the studied species seem to be visible but not very visually attractive to potential pollinators. The food rewards are hidden within the nodding perianth, and both traits are conserved among fritillaries. Additionally, visual floral traits are not good predictors of nectar properties. When in the flowers, pollinators are navigated by nectar guides in the form of contrasting nectary area colouration. Flower colour does not serve as a phenotypic filter against illegitimate pollinators—red and orange bird-pollinated fritillaries are visible to bees.

## Introduction

Visual floral traits are key features that define plants’ interactions with the surrounding environment. As a result, flowers show extreme variation in visual traits, even among closely related taxa^1^. The factors driving this divergence remain an open area of research. The observed diversity of visual floral traits, e.g., colour, size, the arrangement of reproductive parts and reward properties, has largely been attributed to selection mediated by pollinator preferences^2–5^. Among these flower traits, colour plays a crucial role in shaping plant-animal interactions^6^. Numerous studies have shown that flower colour often reflects pollinators’ preferences^7–10^ and plant species pollinated by similar animal groups often share similar colour properties^11^. For instance, in the case of North American *Penstemon* Schmidel species, colour is the best predictor of flower visitation by hummingbirds^12,13^, and in insect-pollinated *Mimulus* L. species, bees responded primarily to tepal colour^14^. The extensive flower divergence may occur because members of different pollinator groups may perceive flowers differently^15–17^ due to variation in their colour vision systems, including the presence or absence of ultraviolet and red light sensitivity.

Attractiveness to potential pollinators is not the only factor shaping flower colour. The risk of losing floral food rewards produced by plants results in selection pressure on flower colour to discourage less effective flower visitors or flower thieves^16^. The most cited examples include ‘anti-bee’ features of hummingbird-pollinated flowers^18,19^, which are described as reflecting mostly long-wavelength light. These traits may decrease their visibility to bees and other illegitimate visitors, helping protect the rewards for legitimate polliatators^7^.

Floral pigments, responsible for flower colour, also play an important role in plant physiology. Therefore, several plant lineages show little or no association between flower colour and pollination mode^20^. In some species, flower colour may be explained by phylogenetic constraints, pleiotropic effects, adaptations against herbivores, pathogens or local habitat conditions^21,22^. Flavonoids, for example, are involved in the plant response to stress caused, e.g., by drought^1^, or protect plants against damage caused by UV radiation^23^.

Similarly to flower colour, flower size is closely linked to pollinator attraction. Larger flowers are more easily detected^24^ and offer more rewards, such as pollen and nectar^25^. As a result, several studies have documented pollinator-mediated phenotypic selection for larger flowers^26^. On the other hand, there are several factors selecting for smaller flowers^24^. Overall, biotic factors other than pollinators (e.g., herbivores), together with abiotic factors (e.g., resource availability and climate), can exert selection for smaller flowers. Theory predicts that smaller flowers may be advantageous, especially in terms of water balance, but only when pollinators are abundant and efficient^24^.

Both of these traits, flower colour and size, in addition to being involved in attraction and protection, may also be a signal informing potential pollinators about the properties of the available reward^27,28^. Nectar and pollen are the most common food rewards offered to pollinators. Of these, nectar is perhaps the most important^29,30^, playing a key role in plant reproduction by rewarding floral visitors^29^. Nectar properties, such as concentration and volume, can determine plant-pollinator interactions^27,31^. Pollinators preferentially visit flowers richer in rewards, which they assess based on visual floral traits^32,33^. Recent studies have also shown the potential role of presenting the reward itself^34^, with pollen or nectar shaping flower attractiveness^35^. The presentation of yellow anthers has been shown to positively affect visitation as well as pollen receipt and pollen removal from flowers^36^. The arrangement of reproductive parts, however, may announce the available rewards but should also “guide” pollinator movements within the flower. Thus, floral traits such as corolla entrance diameter or floral designs likely also evolved to enable and increase the effectiveness of pollination.

Despite the available studies, our understanding of how flowers evolve and adapt to the surrounding environment remains fragmentary. One of the tools used to address questions related to flower evolution is the theory of pollination syndromes^37^. The fundamental assumption of this concept is that flowers adapt to their most efficient functional pollinator group (most efficient in terms of removing and depositing pollen, Dellinger^38^ and literature cited therein). A recent analysis of Dellinger^38^ showed that one can predict pollinators based on flower colour; however, other traits, such as the reward and perianth width, are more reliable. Nevertheless, since numerous plant species are pollinated by multiple functional groups of animals, questions about the influence of such multi-pollinator systems remain open. Furthermore, flower colour shifts, which may result from a single gene mutation, can also lead to a change in other floral traits, such as flower size or reward properties^39^.

To better understand the role of visual floral traits, we studied colour properties and visual signalling in flowers of the temperate spring flowering genus *Fritillaria* (Liliaceae). The choice was based on the variation in visual floral traits and pollination biology and ecology^40–42^. The genus *Fritillaria* comprises approximately 140 species of bulbous plants dispersed predominantly in temperate Holarctic regions of both the Old and New Worlds^43–45^, with the highest diversity observed in the Mediterranean region^46–51^. Flowers of fritillaries are generally nodding, with a tulip-like trimerous perianth concealing nectaries and stamens, usually shorter than the tepals. Several representatives of this genus have unusual tessellation on the tepals formed from a repeating bi-coloured chequered pattern. Despite many similarities, fritillaries differ, e.g., in terms of size and colour (for human vision). The most recent common ancestor, for example, probably had purple flowers, and this colour is the most common among modern species. There was, however, one transition to red flowers and one to orange flowers^52^. We still do not fully understand this divergence in fritillaries, but it may be related to pollination systems, at least to some extent. Flower features of most of the fritillaries indicate pollination by insects, but there have been at least two shifts from insect to bird pollination. Orange Asian *F. imperialis* is pollinated by passerines^53,54^, and red American *F. gentneri* and *F. recurva* are pollinated by hummingbirds^55^. Additionally, the properties of nectar reflect the preferences of their bird pollinators, with hummingbird-pollinated species producing very large amounts of nectar with medium sugar concentrations and passerine bird-pollinated species producing very large amounts of highly diluted nectar^38,41^. Additionally, among insect-pollinated fritillaries, there are possible shifts in the main pollinators. *Fritillaria camtschatcensis* is pollinated by flies^56^, and this species has distinct floral traits and reward properties^40,41^ compared to those of species, e.g., bee-pollinated fritillaries.

Since previous studies have shown that fritillaries offer a robust food reward to pollinators, we wanted to study the visual properties of fritillary flowers and the potential correlation between floral traits and reward properties. How attractive are *Fritillaria* flowers to potential pollinators, and are there any floral guides in the ultraviolet spectrum? Can flower size, i.e., entrance diameter and tepal length, play a role in potential advertisement of the nectar reward? To assess the attractiveness of fritillaries, we determined the areas of the tepals, which are conspicuous to pollinators and thus function as a visual attractant. Subsequently, we measured the spectral reflectance of the inner and outer surfaces of the tepals. Searching for potential nectar guides on the tepals, we measured the reflectance of the nectaries. Additionally, to find out more about the way bee pollinators perceive *Fritillaria* flowers, we prepared false-colour pictures. Finally, we tracked the evolution and assessed the ancestral states of flower features, which may be crucial in shaping plant-pollinator interactions.

## Materials and methods

### Spectral analysis

Most *Fritillaria* species are rare in nature and grow in hardly accessible, remote places. Therefore, performing studies in natural habitats on a satisfying number of species is often not possible. Nonetheless, to gain better insight into plant-pollinator communication in fritillaries, we decided to use material available at the University of Warsaw Botanic Garden (hereafter BG) and in private collections. For this analysis, we used flowers from plants cultivated in the BG and in private collections of Colin Everett (Somerton, Somerset, UK; hereafter CE), Laurence Hill (Richmond, Surrey, UK; hereafter LH) and Paweł Kalinowski (Szczeglacin, Korczew, Poland; hereafter PK). One species (*F. messanensis*) was analysed in situ on Crete. Since most of the *Fritillaria* species are also rare in cultivation, the number of flowers analysed varied due to the availability of plant material (the sources and sample sizes used in this study are listed in Supplementary materials Table 1).

**Figure 1:**
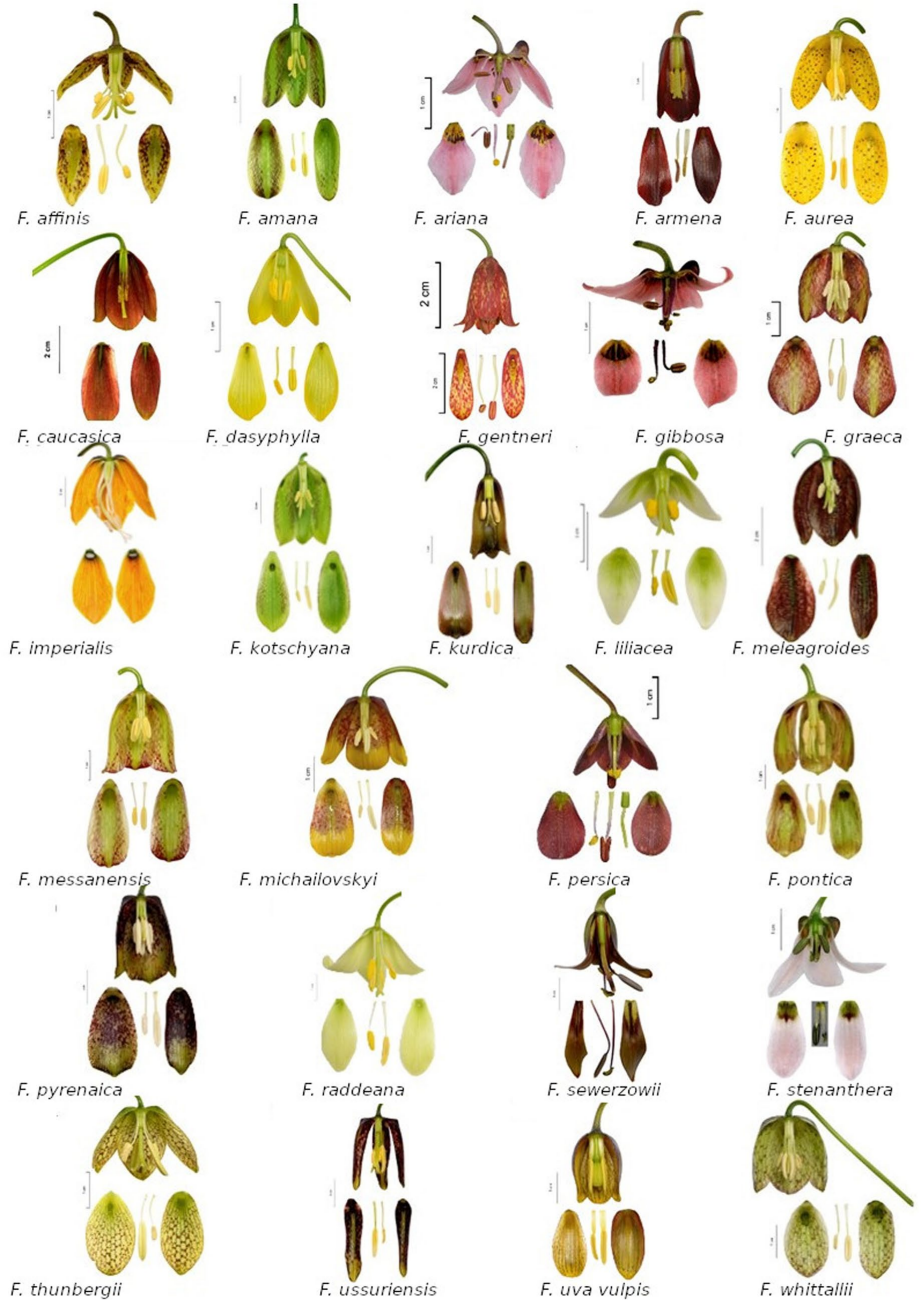
Flowers of some *Fritillaria* species presented in the study (pictures by LH). The images were produced using GNU Image Manipulation Program (GIMP) 2.99.4 (https://www.gimp.org).

To better understand the visual attractiveness of *Fritillaria* flowers, we performed spectral reflectance analyses of 35 species (Fig. 1, Supplementary materials Table 1). Since there are few species of fritillaries with red flowers (with the exception of *F. eastwoodiae,* where the red tepals have a large amount of yellow admixed in) and all of them are bird-pollinated (Supplementary materials Fig. 1), we wanted to test the eventual deterrence of insect visitors by these red flowers, which was previously detected in studies of bird-pollinated species^57^. Flowers from the BG collection were sampled directly prior to analysis. Flowers from the collections of CE, LH and PK were collected and stored in a refrigerator (4 °C) for three to five days before analysis. Storing flowers at a low temperature may potentially change the spectral reflectance of collected specimens, yet to the best of our knowledge, this is one of most common ways of preserving flower colours.

For the collected flowers, we measured the reflectance of all the dominant-coloured areas of the tepals (the most widespread colours within a flower, with the assessment of colour based on human perception) inside and outside of the flowers. Additionally, reflectance was measured for the nectaries (scheme showing the analysis pattern in Supplementary materials Fig. 2). The reflectance curves represent a mean value of five measurements from one flower (prepared using Python version 3.7).

The reflectance measurements were performed with a JAZ Spectrometer System (Ocean Optics Inc., Florida, USA) at an angle of 45° to the measurement site (connected via a fibre-optic cable; UV–VIS 400 μm; World Precision Instruments, Inc., Florida, USA). To calibrate the spectrometer, we used a black standard (black PTFE powder, Spectralon diffuse reflectance standard SRS-02-010, reflectance factor of 2.00%, Labsphere, Inc., North Sutton, USA) and a white standard (white PTFE powder, Spectralon diffuse reflectance standard SRS99-010, reflectance factor of 99.00%).

To analyse the spectra according to a potential pollinator's visual system, we used the *vismodel* function in the ‘pavo’ package. This tool enabled us to show the reflectance spectra in combination with colour-space models of pollinator vision (settings of the *vismodel* analysis in Supplementary materials S1). We analysed the visual systems of bees (*Apis mellifera*), flies (*Musca domestica* and *Eristalis tenax*)*,* butterflies (*Pieris rapae*) and birds (*Cyanistes caeruleum*, a passerine bird, and *Sephanoides sephaniodes*, a hummingbird). These species were selected as representatives of potential pollinator groups based on the literature^41,42,53–56^. Prior to analyses, the reflectance data were smoothed, and the negative values were set to 0 (*procspec* function in the ‘pavo’ package).

To find out more about bee vision and test for the presence of nectar guides in fritillaries, we took pictures with several filters (for details, see below). Subsequently, we prepared false-colour pictures of *Fritillaria* flowers. Many details of fritillary flowers, such as colour patterns (produced by chequered tepals), nectaries, and stamens, are too finely scaled for spectral reflectance measurements. We applied false-colour photography to reveal these colour patterns. These analyses were performed for 34 *Fritillaria* species (Fig. 1, Supplementary materials Table 1). Photographs of the flower details were taken by using a UV-sensitive Nikon D60 (Tokyo, Japan) camera, which was then combined with a filter transmitting human-visible light (UV/IR cut filter: Baader UV-IR-Cut/L-filter) or a UV-transmittance filter (Baader U-filter 2, transmission peak wavelength 350 nm, half-bandwidth 60 nm; Baader Planetarium, Mammendorf, Germany), in daylight. The false-colour images of *Fritillaria* flowers were prepared in Photoshop (version CS5) according to the procedure described in Luanau and VerhoeVen^58^.

### Phylogenetic tree

To understand the evolutionary tendencies of the floral traits of fritillaries, we constructed a phylogenetic tree for phylogeny-based analysis. We used the nuclear ITS (DNA internal transcribed spacer) and the plastid genes *matK* and *rpl16*. We used *Lilium* L. as an outgroup based on the established relationship^43,49^. All of the gene sequences were acquired from GenBank (accession numbers in Supplementary Materials S2) and downloaded using the MatPhylobi program^59^, which is a command-line tool for constructing taxonomic data sets for phylogenetic inference based on NCBI data. To create the phylogenetic database in MatPhylobi, we seeded *F. michailovskyi* and *L. regale* as representatives of the studied genera. The sequences downloaded represent 113 *Fritillaria* species.

All three types of sequences were independently aligned using the online implementation of the multiple alignment program MAFFT (version 7.64^60^). Subsequently, all alignments were imported into a modular system for evolutionary analysis, called Mesquite (version 3.6^61^), for visual inspection. Poorly aligned positions and divergent regions were eliminated using TrimAl (version 1.3, http://phylemon2.bioinfo.cipf.es). The trimmed alignments were then concatenated with RAxML, a tool for phylogenetic analysis (version 8.0^62^).

Using the combined data set, we estimated the divergence time of *Fritillaria*. To do so, we used a log normal relaxed clock in BEAST 2 version 2.6.3^63^. On the basis of the jModelTest results, we used GTR as the substitution model. We specified the tree prior model as the fossilized birth–death model and calculated the posterior distributions of internal nodes with Markov chain Monte Carlo (MCMC) analyses of 10 million generations with a 10% burn-in. To calibrate the tree, we used four dated fossils for *Liliales* previously used in *Fritillaria* dating^64^: *Luzuriaga peterbannisteri* (age range: 23.03–15.97 Ma; in our study, we set the age to 20 Ma), *Smilax labidurommae*^65^ (age range: 37.2–33.9 Ma; in our study, we set the age to 35 Ma), *S. pristina*^66^ (age range: 55.8–48.6 Ma; in our study, we set the age to 51 Ma), and *Ripogonum tasmanicum*^67^ (age range: 55.8–48.6 Ma; in our study, we set the age to 51 Ma). We set the mean and standard deviation of these distributions to 1 and 1.25, respectively, and the confidence interval to 10 Ma.

Using our refined database of sequences, we also used RAxML to generate our final phylogenetic tree for ancestral state analysis. RAxML uses a series of maximum-likelihood (ML) tests to generate the tree. To find the best phylogenetic tree, we used a bootstrap analysis with 1000 replicates for each of the partitions (with models recommended by PartitionFinder^68^).

### Data collection—nectar properties, floral traits, and species distribution

To better understand plant-pollinator communication in *Fritillaria,* we prepared a database describing the diversity of nectar properties and several floral traits that are potentially important for increasing attractiveness in the studied genus. Our sources were scientific literature describing fritillaries, regional floras, and internet sources (http://www.fritillariaicones.com, http://www.efloras.org, and http://www.fritillaria.org.uk). Supplementary materials Table 1 presents all the data. We assessed available information for nectar properties and six floral traits for 113 species present on the phylogenetic tree.

Nectar properties, i.e., volume and sugar concentration, were obtained from Roguz^38,41^. In that study, the authors measured the volume of nectar collected from flowers during anthesis. Flowers for nectar sampling were bagged with mesh during the bud stage to prevent visits by insects. During anthesis but before anther dehiscence, nectar was sampled. All samples assigned to a specific treatment were analysed with the use of the same methods. Sample size varied depending on the species, with a range from four to 70 (Supplementary materials Table 1).

All the studied fritillaries produce nectar, which is easily accessible to pollinators; however, nectaries are usually hidden within the nodding perianth. We assessed the number of species where flowers are not facing down and wide open with visible nectaries. Additionally, we counted species where the nectary presents contrasting colours. Several pollinators visit flowers mainly for pollen^61^; therefore, their presentation may play a crucial role in luring potential pollinators. Therefore, we calculated the number of *Fritillaria* species with anthers visible from a distance, i.e., flowers with a wide open perianth (e.g., *F. bucharica*) or with the anthers exerting beyond the perianth (e.g., *F. imperialis*). *Fritillaria* is one of very few genera where a chequered pattern covers the tepals, so we also collected information regarding the presence or absence of this pattern on the tepals. To estimate the visual attractiveness of the flowers, we collected information about the size of the flowers, i.e., entrance diameter (entrance to the flower at the widest point) and the length of the tepals.

To assess the differences among *Fritillaria* species, we collected information about the distributions of modern representatives of this genus. We defined five distributional areas, namely, North Africa, Asia-temperate, Asia-tropical, Europe, and North America, and a combination of these distributions.

### Phylogenetic studies

Next, using the obtained tree and trait databases, we determined the ancestral states of reward presentation, the chequered pattern, and the species distribution. The analysis was performed using single rate model reconstruction, which gives empirical Bayesian probabilities (‘ape’ package, version 5.0^69^). First, we determined the appropriate transition probability model, choosing among ER—equal rate, SYM—symmetrical rate, and ARD—all-rates different, using a log likelihood ratio analysis. In all cases, the ARD transition ratio model was chosen because it had the highest likelihood value. Then, to compute the total number of character changes, as well as the number of character changes between all states, we used the make.simmap function in the ‘phytools’ package (1000 simulations across the tree^70^).

Finally, using the phylogenetic tree of *Fritillaria,* we tested for correlations between continuous traits and nectar properties (nectar volume, sugar concentration, entrance diameter and tepal length) and discrete traits (anther and nectary presentation, colour of the nectaries, presence of the chequered pattern and species distribution). We conducted phylANOVA on other visual traits (performed in R using the *phylANOVA* function in the ‘phytools’ package). This function performs the simulation-based phylogenetic ANOVA of Garland and Jones^71^. The level of correlation between the continuous traits was assessed by calculating the phylogenetic generalized least squares (PGLS). In this study, the structure of the phylogenetic signal was controlled by the lambda parameter. The branch length transformation was optimized between bounds using maximum likelihood (‘caper’ package, version v1.10.1^72^). The statistical analyses were performed with the statistical computing software R 3.5.3^73^ (R analysis code accessible in Supplementary materials S3).

## Results

### Spectral analysis

**Figure 2:**
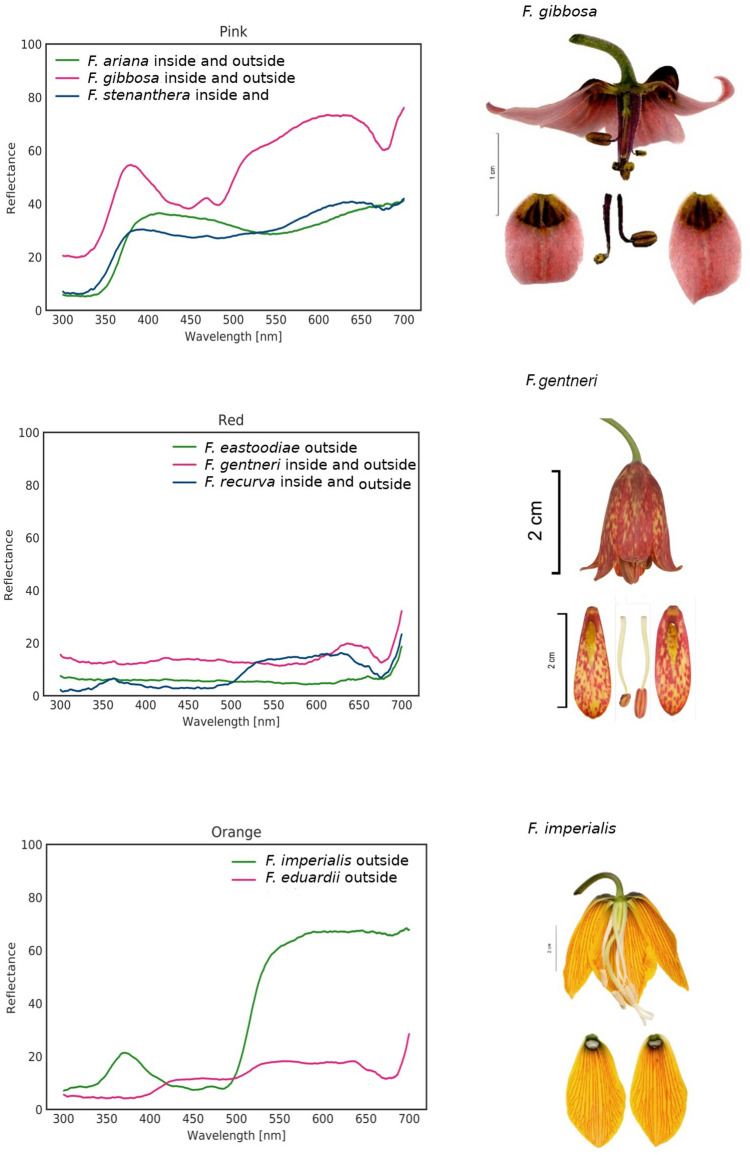
Reflectance of *Fritillaria* flowers of the same colour category representing different subgenera and exemplary species representing the colour category. Species with bright flowers (pink) have high reflectance above 350 nm. Reflectance of the nectary area was much lower than that of tepals for most of the studied *Fritillaria* species. The images were produced using GNU Image Manipulation Program (GIMP) 2.99.4 (https://www.gimp.org).

**Figure 3:**
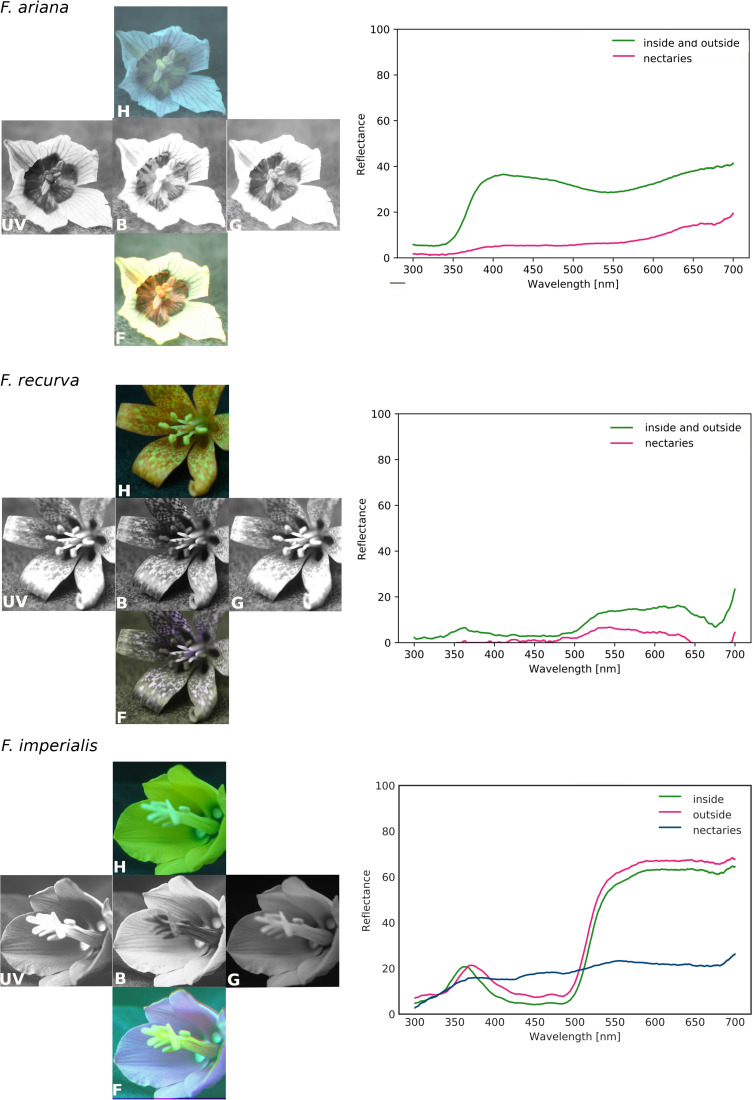
Colour photograph (human vision; H); UV (UV), blue (B), and green photographs (G); false-colour pictures (bee vision; F); and reflectance curves for selected *Fritillaria* species. *Fritillaria ariana* displayed a UV bull’s eye pattern (UV-reflecting centre of the flower) and had relatively high reflectance in the UV range. American *F. recurva* and *F. gentneri* had UV-reflecting white dots in the chequered tepal pattern. *F. imperialis* had UV-absorbing flowers with the exception of UV-reflecting stigmas. Reflectance of the nectary area was much lower than that of the tepals for most of the studied fritillaries. The images were produced using GNU Image Manipulation Program (GIMP) 2.99.4 (https://www.gimp.org).

For most of the studied fritillaries, the reflectance was higher above 500 nm and lower in the blue and UV wavelength range. Species with bright flowers (white or pink) were an exception, with reflectances higher above 350 nm. The ultraviolet reflectance properties of *Fritillaria* species varied within subgenera, with no obvious pattern of UV reflection. For example, the subgenus *Fritillaria* includes UV-reflecting species (*F. montana*) and UV-absorbing species (*F. graeca*). Closely related *F. imperialis* and *F. eduardii,* representing the subgenus *Petilium*, had UV-absorbing tepals and UV-reflecting stigmas (Fig. 2; Supplementary materials Fig. 3). Flowers of *Fritillaria ariana* displayed a UV bull’s eye pattern with a UV-reflecting centre. Additionally, flowers of *F. liliacea* had a UV-absorbing centre. The species with chequered tepals had UV-reflecting white dots (Fig. 3).

For almost all *Fritillaria* species, the reflectance of the nectaries was much lower than that of tepals. Only the nectaries of *F. eastwoodiae*, *F. dasyphylla* and *F. minima* had reflectances similar to or higher than the values recorded for the tepals. The reflectances of the inner and outer sides of the tepals were similar (Figs. 2, 3; Supplementary materials Fig. 3).

*False-colour* pictures indicated that the nectary area in fritillaries is distinctive. As a result, nectaries may act as visible nectar guides for bees. The anthers and pollen grains, mostly yellow under human vision, proved to be visible to bees in both insect- and bird-pollinated flowers. The chequered pattern present on the tepals of some species was also visible to bees. The nectaries of the subgenus *Petilium* had an accumulation of starch, producing a white appearance, which was also confirmed in the *false-colour* pictures (Fig. 3). Bird-pollinated species of fritillaries, i.e., *F. imperialis, F. gentneri* and *F. recurva*, are visible to pollinators, with distinctive anthers and nectary areas.

**Figure 4:**
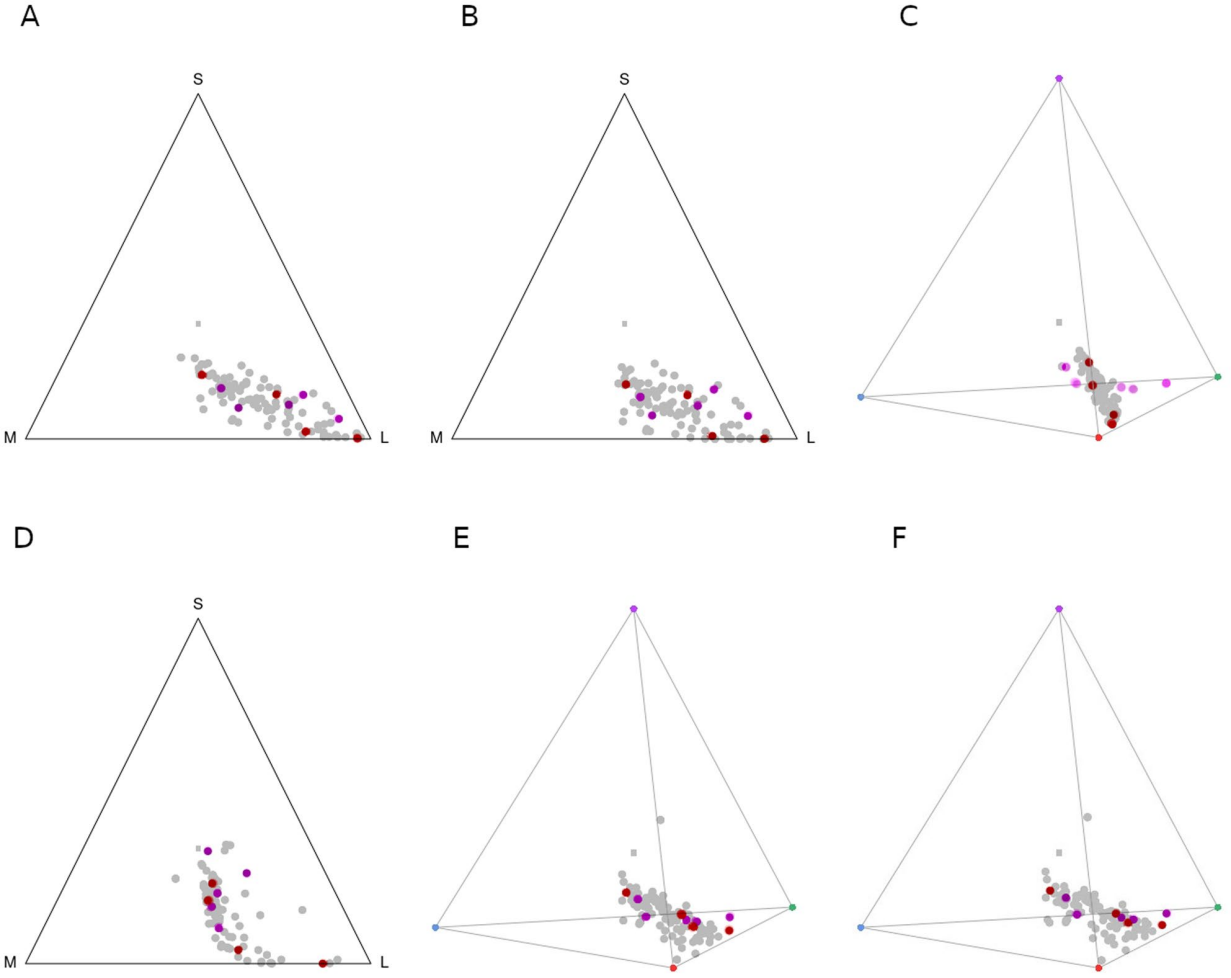
Colour loci in a CH model, where the triangle tops represent the wavelength: S—short, M—medium and L—long. Colour loci are shown in the tetrahedron colour spaces. The inverted triangles in the centres refer to the origin points; purple, blue, green and red vertices represent the maximum signals (relative quantum catches). Pink dots represent passerine bird-pollinated species, red dots represent hummingbird-pollinated species, and grey dots represent the rest of the studied species. Models for bees ([**A**] *Apis millifera*), flies ([**B**] *Eristalis tenax,* [**C**] *Musca domestica*), butterflies ([**D**] *Pieris rapae*)*,* and birds ([**E**] the hummingbird *Sephanoides sephaniodes* and [**F**] the passerine bird *Cyanistes caeruleus*). The images were produced using GNU Image Manipulation Program (GIMP) 2.99.4 (https://www.gimp.org).

Modelling of fritillary flower colour appearance in the chromaticity diagram of *Apis mellifera* and *Eristalis tenax* showed that the studied species densely occupied the portions of long waves: the centre and around the corner with long wavelengths. Bird-pollinated *Fritillaria* flowers occupied the same sections. In the case of *Musca domestica,* the model also showed a large accumulation of species presumably pollinated by insects in the corner representing long waves. Species pollinated by passerine birds were located closer to the green photoreceptor type. For Lepidoptera, represented by *Pieris rapae*, all fritillaries occupied mostly the centre of the diagram. For potential bird pollinators, both representatives of hummingbirds and passerine birds, mostly possessing cones with long- and medium-wavelength sensitivities, were stimulated. The accumulation of most points in the lm range suggested the stimulation of cone oil droplets with carotenoid filters (Fig. 4).

### Flower traits, nectar properties, pollinators, and species distribution

All the studied *Fritillaria* species produced nectar, which is an important reward for pollinators. Its concentration and volume were variable, ranging from 5.3 to 64.9% and from 0.3 to 205 µl per flower, respectively.

The flowers were usually bell-shaped and nodding, and as a result, nectaries and anthers were not visible to approaching animals. In general, 13.9% of fritillaries (16 species) had nectaries visible from the outside, and 21.7% (25 species) had anthers that may be perceived from a distance. The nectaries usually had different colours than the background (75.6%, 87 species). The chequered pattern was common among the *Fritillaria* species, where 54% (61 species) had spotted tepals. The perianth entrance diameter and the length of the tepals were variable and depended largely on the species, with ranges from 9.3 to 67.8 mm and 10.5 to 53.2 mm, respectively.

### The relationships among the studied features—phylogeny-based analyses

**Figure 5:**
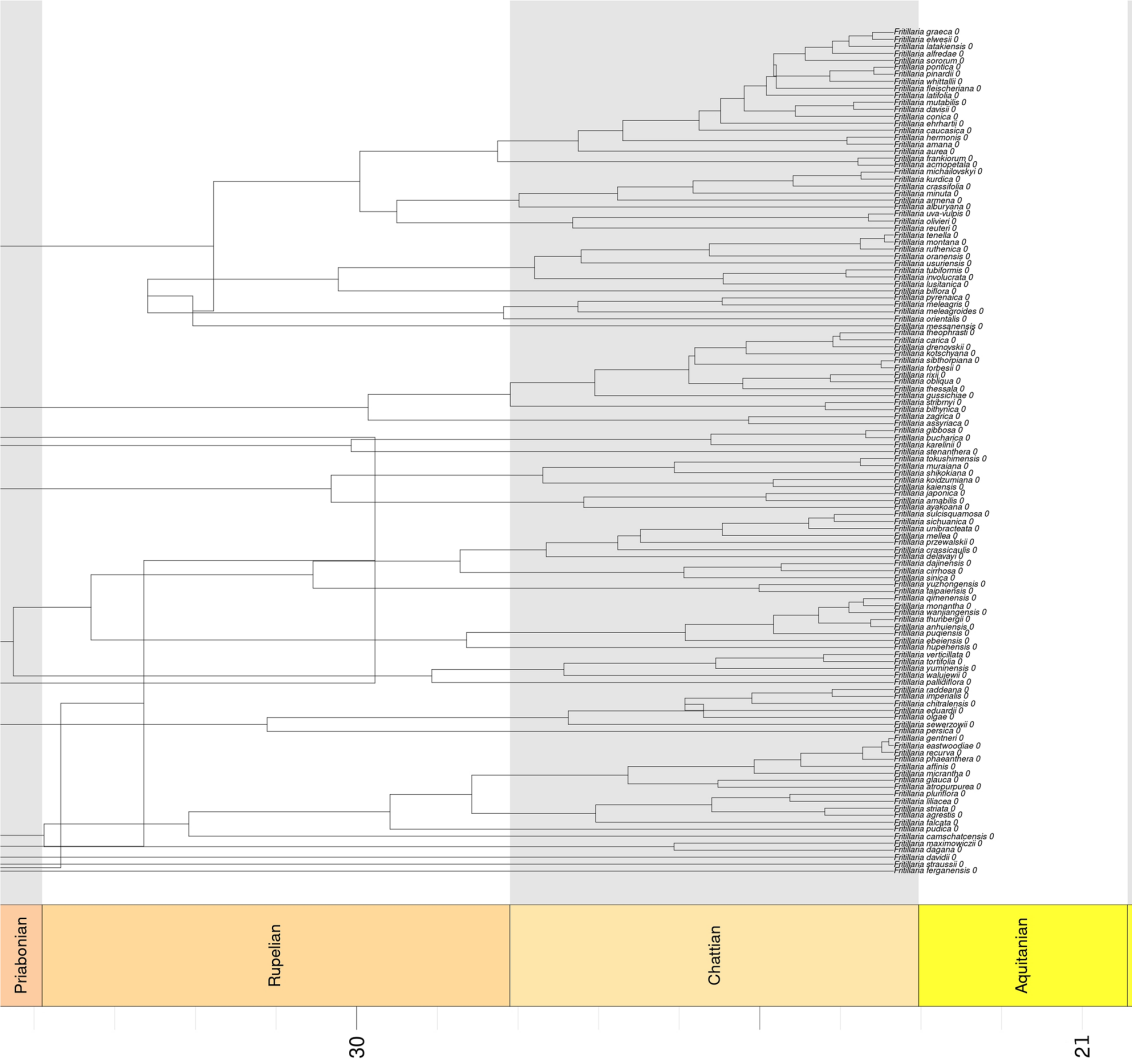
The maximum clade credibility tree of *Fritillaria* summarized by TreeAnnotator and plotted with a geological timescale using the strap package geoscalePhylo function in R. The timeline at the bottom indicates the age (Mya, million years ago) of nodes.

*Fritillaria* was estimated to have originated approximately 38 Mya, in the early Oligocene (Fig. 5). The most recent common ancestor of *Fritillaria* occurred in temperate Asia, where the highest diversity of fritillaries is still found (Supplementary materials Fig. 4). Expansion to North America occurred early in the evolution of this genus. Species pollination by passerine birds evolved earlier (approximately 27.5 Mya) and hummingbird-pollinated species appeared recently in the phylogeny of the studied genus (approximately 24 Mya).

The ancestral state reconstruction in *Fritillaria* suggests that the most recent common ancestor probably had flowers with nectaries hidden deeply within the perianth but with a distinctive colour (Supplementary materials Fig. 5A,B). Both of these states were also the most common across the phylogeny and among modern species (Supplementary materials Table 2). The anthers of most fritillaries are and always have been not visible from a distance (Supplementary materials Fig. 5A,B). Even when visible nectaries and/or anthers evolved, these features were lost several times. The chequered pattern also evolved early during the evolution of this genus. The majority of internal nodes had a pattern on the tepals. Several subgenera, such as *Petilium, Theresia* and *Korolkovia* (species representing the subgenera mentioned in Supplementary materials Table 1), appear to have arisen from an ancestor with chequered patterns on the tepals, with recent loss of this character (Supplementary materials Fig. 5A,B). All the studied flower features were labile, with the highest number of changes observed for the presence or absence of the chequered pattern (747 shifts between the states) and the lowest for the visibility of the anthers (56.8, Supplementary materials Table 2).

Taking into account the phylogenetic relationships among fritillaries, we did not find associations between floral traits and nectar properties (Supplementary materials Table 3). Testing for correlations between the studied characters with PGLS, we found only two significant negative correlations: between entrance diameter and tepal length (r = − 0.09, *p *value < 0.05) and between nectar volume and entrance diameter (r = − 0.02, *p *value < 0.05, Supplementary materials Table 4).

## Discussion

The flowers of fritillaries show a wide diversity of colours and colour patterns. In this study, we assess the attractiveness of *Fritillaria* flowers to potential pollinators and search for correlations between visual traits and reward properties. We also track the evolutionary tendencies of floral traits related to reward presentation. Most *Fritillaria* flowers reflect wavelengths > 500 nm, while hymenopteran pollinators are likely to have the best discrimination for wavelengths close to 400 nm and 500 nm^74^. Therefore, the plant-pollinator relationship in fritillaries seems to be based on robust food rewards; however, these rewards are not advertised. Neither flower entrance diameter nor tepal length is correlated with nectar properties. Most of the modern species also have both nectaries and anthers hidden deeply in a nodding perianth, which is also the most likely state of the recent common ancestor.

When pollinators are in the flower, the nectaries are distinctive, and their colours differ from that of the background. Different nectary colour is and has been common among fritillaries. Red and orange flowers are found only among bird-visited fritillaries. The principles of the pollination syndrome theory, however, may be applied to fritillaries only to a limited extent. Flowers of these species are also visited by bees, suggesting ecological generalization, despite some level of phenotypic specialization. One of the reasons for this^75^ may be relatively late diversification. Fritillaries originated approximately 38 Mya, with the most recent common ancestor found in temperate Asia, but specific pollination syndromes appeared later. For example, species pollinated by hummingbirds appeared approximately 24 Mya.

False-colour pictures prepared for fritillaries proved that flower colours of bird-pollinated species and the rewards within were visible to bees. This also confirms observations from natural habitats, where North American red-flowered fritillaries are visited by bees^55^. It is important to note that among fritillaries, an evolutionary transition from purple to red flowers is common^51^. This type of shift often accompanies shifts in adaptation from bee to hummingbird pollination or co-occurs with other floral adaptations that facilitate hummingbird pollination^76^. Several studies proved the attractiveness of red-flowered species to insects^77–79^; however, to protect the reward, some hummingbird-pollinated flowers reflect mostly long-wavelength light (median wavelengths > 585 nm), as this decreases their visibility and helps avoid visits by illegitimate pollinators^7,16^. This was not the case for the studied species, which may be related to their relatively recent evolution. Hummingbird-pollinated fritillaries evolved in North America and are absent in Europe and Asia. Due to this relatively recent evolution, hummingbird-pollinated fritillaries probably had no chance to adapt to these birds in the Old World^80,81^.

Similarly, as in the case of hummingbird-pollinated flowers, passerine bird-pollinated fritillaries are also visible to insects. Flowers of these species also have distinctive colouration within the genus: typically, orange-red (rarely yellow) flowers that lack reflectance in the UV range. As with hummingbird-pollinated North American fritillaries, it is still unclear why Asian bird-pollinated *F. imperialis* and *F. eduardii* exhibit this colouration or if passerine birds have a preference for these colours. We assume that tepal colour is an important long-distance signal^57^, although the rewards reflecting the preference of these pollinators (hexose- and amino acid-rich nectar, lacking sucrose^40,41^ in passerine bird-pollinated flowers and very large amounts of nectar with low amino acid concentrations in hummingbird-pollinated species) are equally important. The observed ecological generalization, despite some level of phenotypical specialization^75^, observed in fritillaries may be a result of relatively short-duration fritillary-bird interactions. Studies testing the evolution of flower colour and rewards in members of this genus suggest that changes in these flower features may have appeared only recently^52^, and Asian *F. imperialis* and *F. eduardii* flowers visited and pollinated by passerine birds^53,54^ probably evolved approximately 27.5 Mya.

Despite the importance of visual floral traits in shaping plant-pollinator interactions^82^, we did not find visual attractants described as important for insect pollinators^83–85^ in most of the studied species. For example, our analyses indicated overall UV absorbance and did not indicate additional ultraviolet-reflecting areas. UV-reflecting flower parts are common among insect-pollinated species^7,86^, e.g., in yellow bee-pollinated flowers^86,87^, where they form the so-called bull’s eye pattern with a UV-reflecting periphery and UV-absorbing centre^88^. In fritillaries, we detected the bull’s eye effect in species with bright pink flowers, namely, *F. ariana*, *F gibbosa* and *F. stenanthera.* These closely related species have wide open, erect flowers; therefore, we assume that the angle of the flower may correlate with the presence of the bull’s eye pattern—it could not be seen in downward-facing bell-shaped flowers. The lack of a strong visual signal, common among fritillaries, is most likely caused by the thick wax cover present in almost all fritillaries^41^. Additionally, the similarity of the reflectance curves found in our study may be due to the similarity of tepal ultrastructure in *Fritillaria,* which may determine the reflectance amplitude^89^. Previous studies conducted by Roguz^39^ revealed that almost half of the genus has nearly the same tepal and nectary ultrastructure.

We assume that in fritillaries, where the reflectance does not fit the preferences of the most important pollinators, visual signals perceived from a short distance may play an important role. The presence of the chequered pattern, although labile, evolved early in fritillary diversification and is very common among modern species. The chequered pattern, rare among other plant species, can enhance flower attractiveness^90^ by increasing the contrast between the object and background through motion parallaxis^91^. Such purple-yellow mottled tepals, common among fritillaries, can also increase the attractiveness of their flowers to flies. These insects are pollinators of at least one fritillaria species (*F. camschatcensis*^56^) and have been seen visiting several other species (authors’ observations).

Additionally, nectaries and anthers, hidden deeply within a nodding perianth, are common and conserved states. In most of the fritillaries, the placement of the reward is “highlighted” by nectaries in different colours, and contrasting nectaries evolved early and are frequent among modern species. Previous studies on *Fritillaria* revealed a lack of phylogenetic signal for nectar properties. These traits show high variability at the community level, which might suggest adaptive evolution in response to different environmental conditions^27^. These variabilities in *Fritillaria* nectar volume and concentration may also be a reason for the lack of correlations between floral traits and nectar properties. These results, however, are surprising, since several studies have shown an association between flower size and rewards^27,32,92^. Nevertheless, the most luring advertisement is to display the reward itself, which then confers the dual role of both signal and reward^93,94^. Open flowers with nectar displayed in the form of large droplets and bright yellow pollen contrasting with the dull flower evolved in *Fritillaria* only a few times. Both of these traits were lost several times across the phylogeny.

Although plant pollinators are among the most important factors influencing visual floral traits, their variability may also be maintained by selection related to environmental heterogeneity. Two of the most common colours in the case of *Fritillaria* flowers are green and purple, which may be related to random or non-adaptive processes maintaining flower colour variation^95^. However, this colour polymorphism may also be related to the ecological role of the pigments responsible for this type of colouration. Studies by Bazzaz and Carlson^96^ revealed, for example, that the photosynthetic activity of green flowers may be enough to maintain their own structure. On the other hand, floral pigments such as anthocyanins, related to violet–purple colouration, function in response to plant stress caused, e.g., by drought, cold or nitrogen deficiency.

## Conclusions

The attractiveness of *Fritillaria* flowers appears to be linked to early blooming and copious rewards. Flower signalling in *Fritillaria* seems to follow a rather simple pattern, with most flowers having almost uniform displays or occasional coloured peripheral and central parts of the flower. For animals already visiting the flowers, fritillaries have nectar guides in the form of contrasting nectary area colouration. This study and recent works on other aspects of pollination biology in *Fritillaria* indicate that plant-pollinator interactions may be responsible for a significant share of the floral diversity in the genus. The principles of the pollination syndrome theory, however, may be applied to fritillaries with some limitations. Flower colour does not play a role as a phenotypic filter—red bird-pollinated fritillaries are also attractive to bees. The widespread purple and green colouration among *Fritillaria*, however, may potentially indicate some role of the environment in this trait. Clarifying the extent to which flower colour in *Fritillaria* is maintained by active selection pressures from pollinator preference and/or the environment remains an open question.

## Supplementary Information

Supplementary Figure 1.

Supplementary Figure 2.

Supplementary Figure 3.

Supplementary Figure 4.

Supplementary Figure 5.

Supplementary Table 1.

Supplementary Table 2.

Supplementary Table 3.

Supplementary Table 4.

Supplementary Information 1.

Supplementary Information 2.

Supplementary Information 3.

Supplementary Captions.
