# Supplementary figures and images for: Visibility and attractiveness of Fritillaria (Liliaceae) flowers to potential pollinators
Source: Sci Rep. 2021 May 26;11:11006. doi: 10.1038/s41598-021-90140-7 (PMC8155214; doi:10.1038/s41598-021-90140-7)

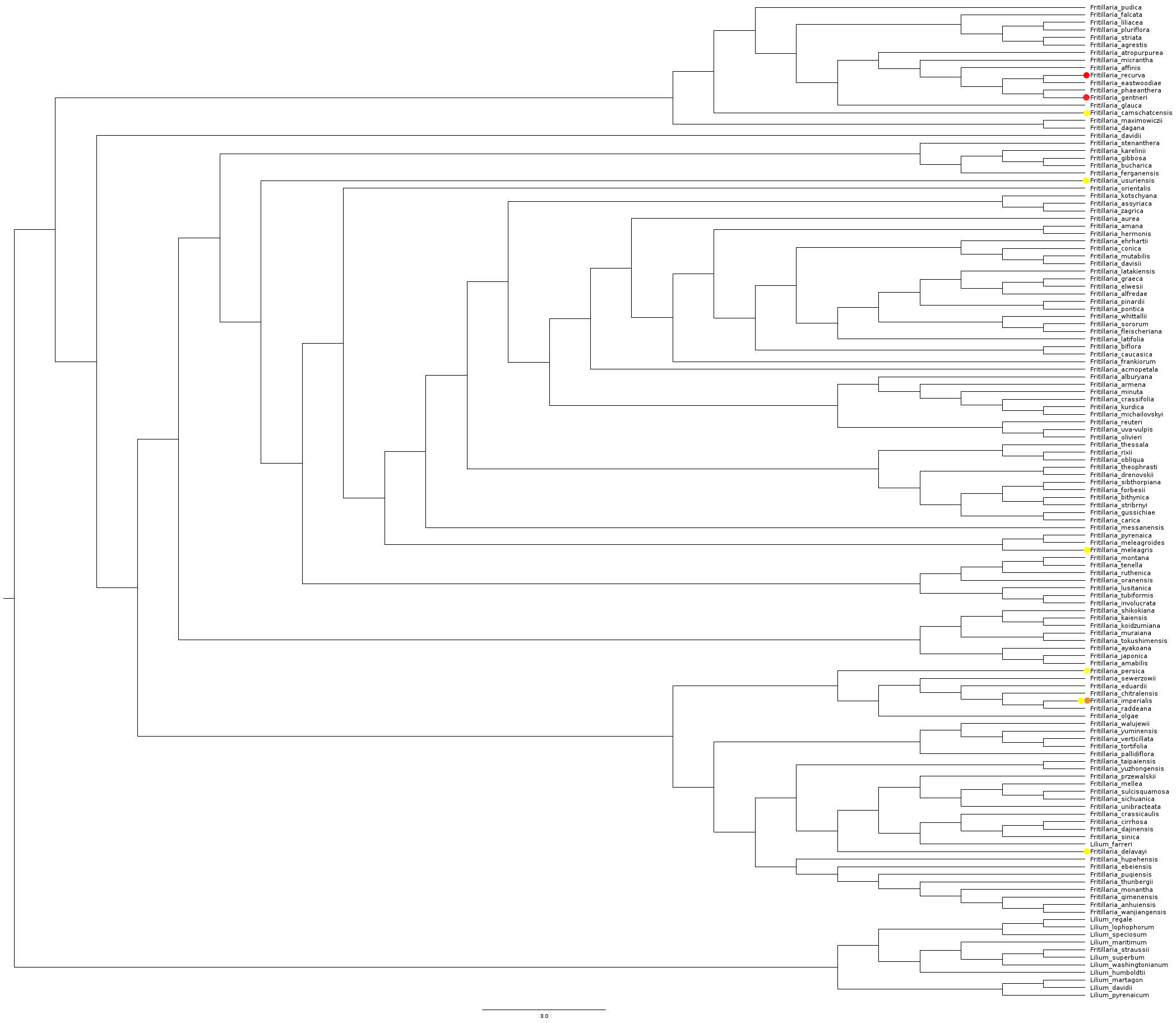

Supplement: Supplementary file 1 — Supplementary Figure 1. [file 41598_2021_90140_MOESM1_ESM.jpg]

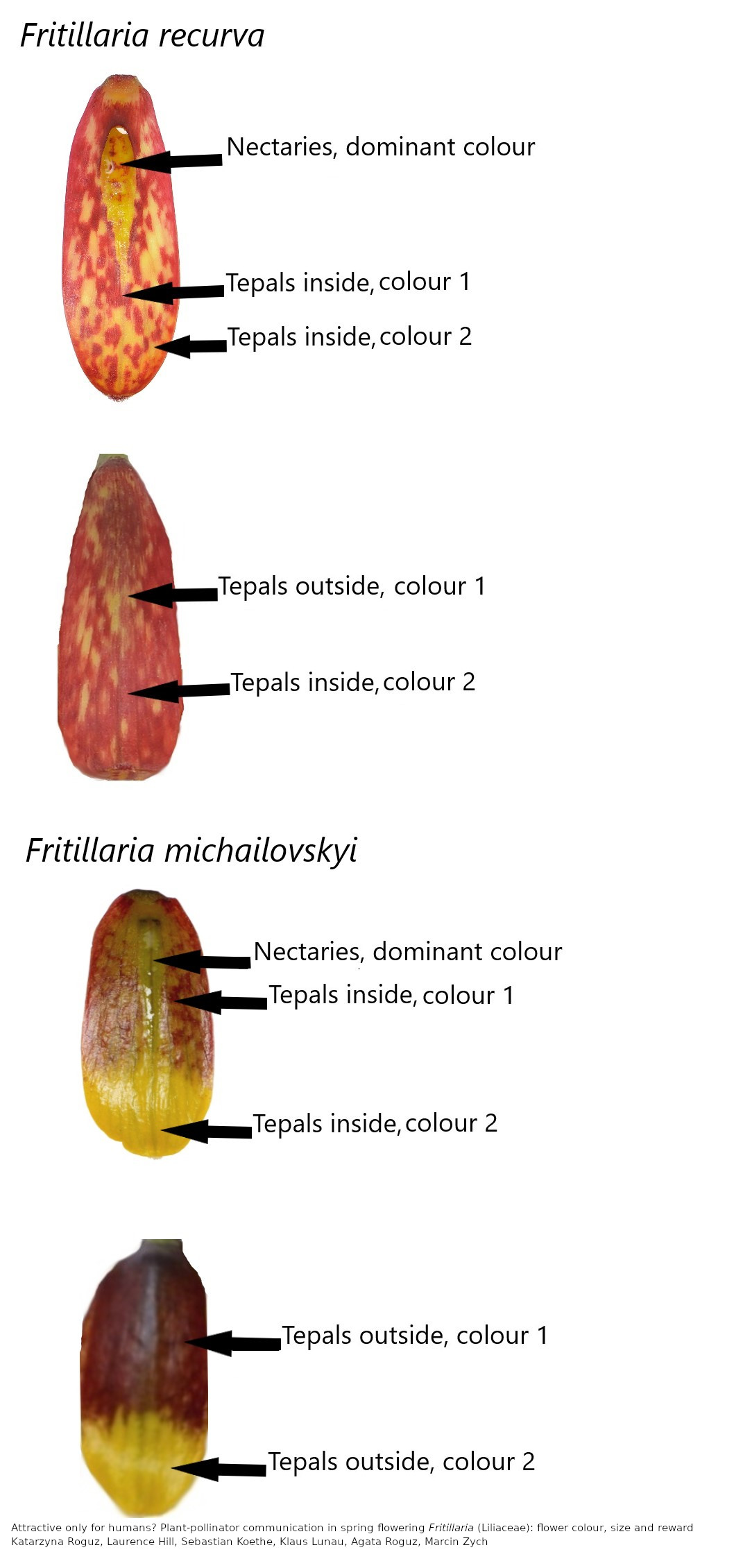

Supplement: Supplementary file 2 — Supplementary Figure 2. [file 41598_2021_90140_MOESM2_ESM.jpg]

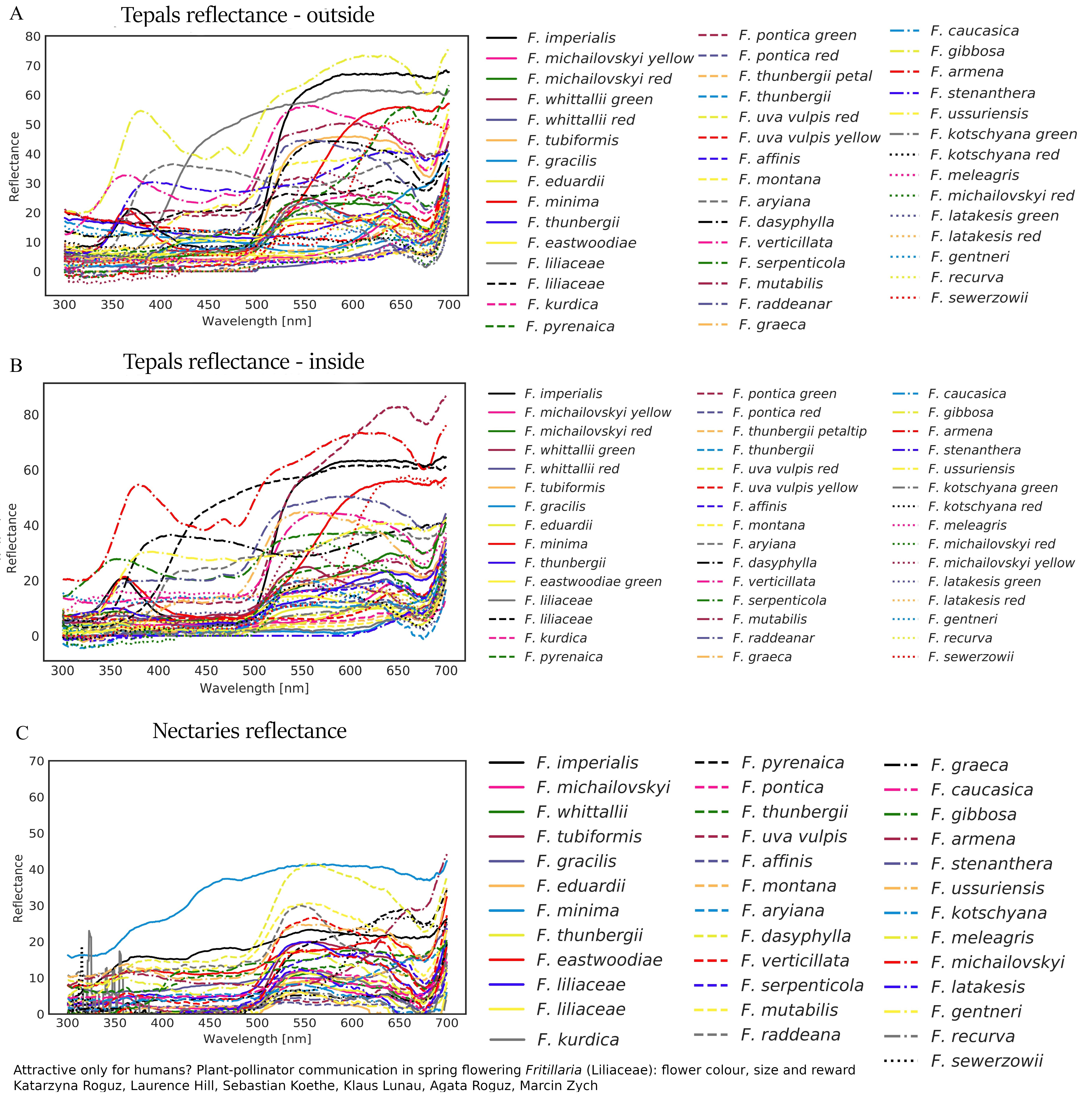

Supplement: Supplementary file 3 — Supplementary Figure 3. [file 41598_2021_90140_MOESM3_ESM.jpg]

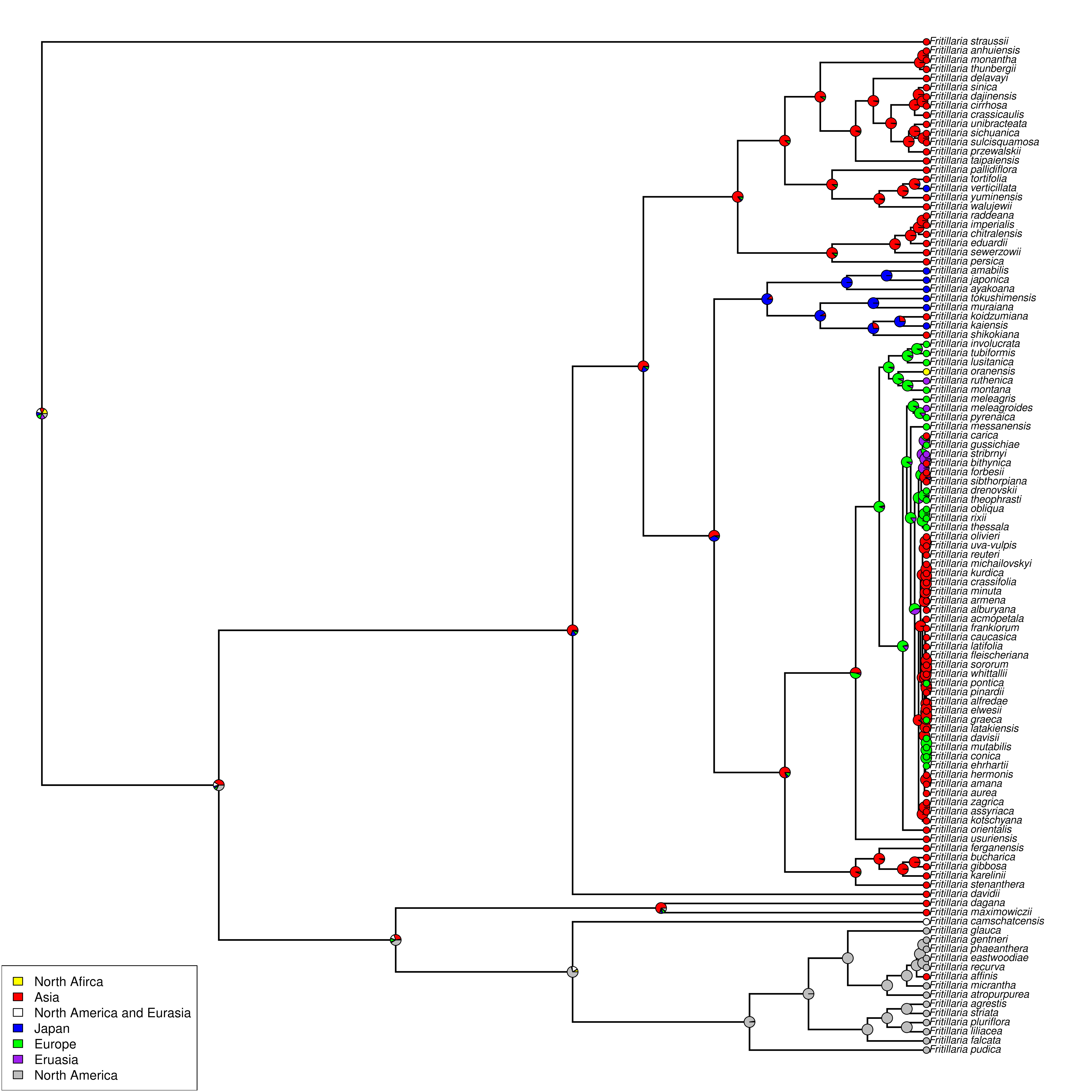

Supplement: Supplementary file 4 — Supplementary Figure 4. [file 41598_2021_90140_MOESM4_ESM.jpg]
