# Supplementary material for: Visibility and attractiveness of Fritillaria (Liliaceae) flowers to potential pollinators
Source: Sci Rep. 2021 May 26;11:11006. doi: 10.1038/s41598-021-90140-7 (PMC8155214; doi:10.1038/s41598-021-90140-7)
Supplement: Supplementary file 7 — Supplementary Table 2. [file 41598_2021_90140_MOESM7_ESM.docx]

|  | Chequered pattern (1) /no chequered pattern (0) | Visible anthers (1)  /no visible anthers (0) | Nectaries with distinctive colour (1)  /nectaries in a background colour (0) | Visible nectaries (1)  /nectaries not visible (0) |
| --- | --- | --- | --- | --- |
| Average number of changes between states  Number of specific transitions | 747 | 56.8 | 464 | 72.3 |
| 1 ->0 | 369 | 28.8 | 232 | 36.3 |
| 0<- 1 | 377 | 28.1 | 232 | 36.1 |
| Proportion of time spent in the state 1 | 0.56 | 0.67 | 0.73 | 0.75 |
| Proportion of time spent in in the state 0 | 0.44 | 0.33 | 0.27 | 0.25 |

Table 2. Results of simulated stochastic character mapping on *Fritillaria* phylogenetic tree (all-rates different selected as transition probability model). Number and time of transitions between studied *Fritillaria* characters identified with the use of make.simmap function in the phytools R package. The estimations are based on 1000 trees with a mapped character.
