# Supplementary material for: Visibility and attractiveness of Fritillaria (Liliaceae) flowers to potential pollinators
Source: Sci Rep. 2021 May 26;11:11006. doi: 10.1038/s41598-021-90140-7 (PMC8155214; doi:10.1038/s41598-021-90140-7)
Supplement: Supplementary file 8 — Supplementary Table 3. [file 41598_2021_90140_MOESM8_ESM.docx]

Table 3. Results of the simulation-based phylogenetic ANOVA assessing the effects of species distribution, anthers visibility, contrasting nectary area, nodding flowers and the presence of chequered pattern on visual flower traits in *Fritillaria* (performed in R using phylANOVA function in phytools package).

|  | species distribution | | | anthers visible from a distance | | | contrasting nectary area | | | nodding flowers | | | chequered pattern | | |
| --- | --- | --- | --- | --- | --- | --- | --- | --- | --- | --- | --- | --- | --- | --- | --- |
|  | Sum Sq | F value | Pr(>F) | Sum Sq | F value | Pr(>F) | Sum Sq | F value | Pr(>F) | Sum Sq | F value | Pr(>F) | Sum Sq | F value | Pr(>F) |
| Tepal length | 697.5 | 1.60 | 0.92 | 114.6 | 1.52 | 0.77 | 62.6 | 0.83 | 0.34 | 479.8 | 6.74 | 0.44 | 210.2 | 2.83 | 0.09 |
| Entrance diameter | 215.7 | 0.21 | 1 | 743.6 | 4.98 | 0.38 | 127.3 | 0.73 | 0.52 | 131.0 | 0.75 | 0.48 | 130.1 | 0.75 | 0.52 |
| Nectar volume | 985 | 0.27 | 0.97 | 1990 | 1.18 | 0.75 | 7.33 | 0.00 | 0.95 | 2501 | 1.51 | 0.46 | 23.1 | 0.01 | 0.95 |
| Nectar concentration | 753.9 | 1.52 | 0.84 | 109 | 0.42 | 0.84 | 60.3 | 0.23 | 0.63 | 1240 | 5.67 | 0.19 | 4.16 | 4.16 | 0.95 |
