# Supplementary material for: Visibility and attractiveness of Fritillaria (Liliaceae) flowers to potential pollinators
Source: Sci Rep. 2021 May 26;11:11006. doi: 10.1038/s41598-021-90140-7 (PMC8155214; doi:10.1038/s41598-021-90140-7)
Supplement: Supplementary file 9 — Supplementary Table 4. [file 41598_2021_90140_MOESM9_ESM.docx]

Table 4. Correlation coefficients and p-values for floral traits (tepal length and perianth entrance diameter) and nectar properties (volume and sugar concentration) for all studied species. Values obtained from calculations of phylogenetic generalized least squares. Bolded correlation coefficients are significant.

| Trait | Entrance diameter | Concentration | Volume |
| --- | --- | --- | --- |
| Tepal length | **-0.09** | -0.11 | -0.02 |
| *p*-value | **0.001** | 0.40 | 0.09 |
| Entrance diameter | | -0.11 | **-0.02** |
| *p*-value |  | 0.08 | **0.04** |
| Concentration | |  | -0.02 |
| *p*-value |  |  | 0.13 |
