# Supplementary material for: Visibility and attractiveness of Fritillaria (Liliaceae) flowers to potential pollinators
Source: Sci Rep. 2021 May 26;11:11006. doi: 10.1038/s41598-021-90140-7 (PMC8155214; doi:10.1038/s41598-021-90140-7)
Supplement: Supplementary file 13 — Supplementary Captions. [file 41598_2021_90140_MOESM13_ESM.docx]

Supplementary materials

Fig. 1. Maximum likelihood tree showing available information about pollinators of

*Fritillaria* flowers (based on the literature). Species marked with a yellow dot are

described as pollinated by insects, and species with a red dot are described as pollinated by insects and

birds. For species with no colour, information about pollinators is missing.

Fig. 2. Diagram showing the *Fritillaria* tepal areas used for spectral analysis. The images were produced using GNU Image Manipulation Program (GIMP) 2.99.4 (https://www.gimp.org).

Fig. 3. Reflectance curves for all studied *Fritillaria* species (A – tepals outside, B – tepals inside, and C – nectaries).

Fig. 4. Estimation of ancestral states of the *Fritillaria* species distribution calculated using maximum likelihood across the posterior distribution. Pie charts represent the proportion of the likeliest state at each internal node. Five defined distributional areas, namely, North Africa, Asia-temperate, Asia-tropical, Europe, and North America, or a combination of these distributions.

Fig. 5. Estimation of ancestral states of flower features among the studied *Fritillaria* species, calculated using maximum likelihood across the posterior distribution. Pie charts represent the proportion of the likeliest state at each internal node. Different pollinator groups (based on the literature) are marked with asterisks: yellow – insect-pollinated, orange – passerine bird-pollinated, red – hummingbird-pollinated. [A] nectary visibility, [B] nectaries with a distinctive colour, [c] anther visibility, [D] chequered pattern presence.

S1. Settings of the *vismodel* analysis.

S2. GenBank accession numbers.

S3. R analysis code
