# Supplementary material for: Visibility and attractiveness of Fritillaria (Liliaceae) flowers to potential pollinators
Source: Sci Rep. 2021 May 26;11:11006. doi: 10.1038/s41598-021-90140-7 (PMC8155214; doi:10.1038/s41598-021-90140-7)
Supplement: Supplementary file 10 [file 41598_2021_90140_MOESM10_ESM.odt]

#Apis mellifera

achrom value: “none”

# Musca domestica: 

achrom value:„md.r1”

#Cyanistes caeruleus

achrom value: “bt.dc”

#Pieris rapae 

achrom value:  “none”

#Eristalis tenax

achrom value:  “none”

#Sephanoides sephaniodes

achrom value:  “none”

The remaining parameters were the same for all studied species, and were set as follows: illum – “D65”, qcatch – “Qi”, vonkries – “YES”, scale -”500”, relative – “TRUE”.
