# Supplementary material for: Visibility and attractiveness of Fritillaria (Liliaceae) flowers to potential pollinators
Source: Sci Rep. 2021 May 26;11:11006. doi: 10.1038/s41598-021-90140-7 (PMC8155214; doi:10.1038/s41598-021-90140-7)
Supplement: Supplementary file 11 [file 41598_2021_90140_MOESM6_ESM.odt]

Table 1. The list of studied Fritillaria species. C – most dominant colours found on the tepals of studied species. Area: A - North Africa, B - Asia-Temperate, C - Asia-Tropical, D - Europe, E - North America, or a combination of these distributions. Studied visual flower traits: ANT – anthers visibility; NEC – nectaries in contrasting colours; REW – reward visible from a distance; PAT – presence of the chequered pattern; ENT – entrance diameter [mm], TEP – tepal length [mm]. Nectar properties: V – nectar volume [μl]; CON – nectar concentration [%]. Source of plants for spectral analyses and false colour pictures: University of Warsaw Botanic Garden (BG); private collections: Colin Everett (Somerton, Somerset, UK; CE), Laurence Hill (Richmond, Surrey, UK; LH) and Paweł Kalinowski (Szczeglacin, Korczew, Poland; PK).

| Species | C | Distribution | Area | ANT | NEC | REW | PAT | ENT | TEP | V | CON | Source of information | Source of plants |
| --- | --- | --- | --- | --- | --- | --- | --- | --- | --- | --- | --- | --- | --- |
| F. acmopetala Boiss. |  |  | Middle East | B | no | yes | no | no | 27.9 | 36.6 | 34.8 | 35.4 | [www.fritillariaicones.com](http://www.fritillariaicones.com/), Roguz et al. 2018. Evolution of bird and insect flower traits in Fritillaria L. (Liliaceae). Frotniers in Plant Sciences |  |
| F. affinis (Schult.and Schult.f.) Sealy |  |  | North America | F | yes | yes | yes | yes | NA | 25 | NA | NA | [www.efloras.org](http://www.efloras.org/), [www.fritillariaicones.com](http://www.fritillariaicones.com/) | BG |
| F. agrestis (Greene) Greene |  |  | North America | F | no | no | no | yes | NA | 26.5 | NA | NA | www.efloras.org |  |
| F. alburyana Rix |  | Turkey | B | yes | yes | yes | yes | NA | NA | NA | NA | [www.fritillaria.org.uk](http://www.fritillaria.org.uk/), Tekşen and Aytaç. 2011. The revision of the genus Fritillaria L. (Liliaceae) in the Mediterranean region (Turkey). Turkish Journal of Botany |  |
| F. alfredae Post |  | Turkey, Syria, Lebanon | B | no | no | no | no | NA | 24 | NA | NA | [www.fritillaria.org.uk](http://www.fritillaria.org.uk/), Tekşen and Aytaç. 2011. The revision of the genus Fritillaria L. (Liliaceae) in the Mediterranean region (Turkey). Turkish Journal of Botany |  |
| F. amabilis Koidz. |  | Japan | C | no | yes | no | no | NA | NA | NA | NA | www.fritillariaicones.com |  |
| F. amana (Rix) R.Wallis and R.B.Wallis |  |  | Turkey | B | no | yes | no | yes | 15.5 | 24.8 | 12.4 | 47.8 | [www.fritillariaicones.com](http://www.fritillariaicones.com/), Roguz et al. 2018. Evolution of bird and insect flower traits in Fritillaria L. (Liliaceae). Frotniers in Plant Sciences | BG |
| F. anhuiensis S.C. Chen & S.F. Yin in S.F. Yin |  |  | China | B | no | yes | no | yes | NA | 40 | NA | NA | [www.efloras.org](http://www.efloras.org/) |  |
| F. ariana Loz.-Lozinsk. & Vved. |  | Central and Western Asia | B | yes | yes | yes | no | NA | 25 | NA | NA | Rix. 2014. 854 Fritillaria ariana. Curtis's Botanical Magazine | LH |
| F. armena Boiss. |  | Turkey | B | no | yes | no | no | NA | 20 | NA | NA | [www.efloras.org](http://www.efloras.org/), www.fritillariaicones.com | BG,LH |
| F. assyriaca Baker. |  |  | Iran, Iraq, Turkey | B | no | yes | no | no | 13.5 | 22.6 | 7.8 | 47.8 | Roguz et al. 2018. Evolution of bird and insect flower traits in Fritillaria L. (Liliaceae). Frotniers in Plant Sciences, Tekşen and Aytaç. 2011. |  |
| F. atropurpurea Nutt. |  |  | North America | F | yes | no | yes | yes | NA | 17.5 | NA | NA | www.efloras.org |  |
| F. aurea Schott |  |  | Turkey | B | no | no | no | yes | NA | 20 | NA | NA | [www.fritillariaicones.com](http://www.fritillariaicones.com/), Tekşen and Aytaç. 2011. The revision of the genus Fritillaria L. (Liliaceae) in the Mediterranean region (Turkey). Turkish Journal of Botany | PK |
| F. ayakoana Maruy. and Naruh. |  | Japan | C | no | yes | no | no | NA | NA | NA | NA | www.fritillariaicones.com |  |
| F. biflora Lindl. |  | North America | F | yes | no | no | no | NA | 29 | NA | NA | [www.fritillariaicones.com](http://www.fritillariaicones.com/), [www.efloras.org](http://www.efloras.org/) |  |
| F. bithynica Baker |  | Turkey | B | no | yes | no | no | NA | 21 | NA | NA | [www.fritillariaicones.com](http://www.fritillariaicones.com/), Tekşen and Aytaç. 2011. The revision of the genus Fritillaria L. (Liliaceae) in the Mediterranean region (Turkey). Turkish Journal of Botany | LH |
| F. bucharica Regel |  | Afghanistan, Tajikistan, Uzbekistan | B | yes | yes | yes | no | 19 | 14.9 | 0.3 | 52.7 | [www.efloras.org](http://www.efloras.org/), Roguz et al. 2018. Evolution of bird and insect flower traits in Fritillaria L. (Liliaceae). Frotniers in Plant Sciences |  |
| F. camtschatcensis (L) Her Gawl. |  |  | Alaska, Rosja | BF | yes | no | no | no | 18.7 | 21.7 | NA | NA | [www.efloras.org](http://www.efloras.org/), [www.fritillariaicones.com](http://www.fritillariaicones.com/), Roguz et al. 2018. Evolution of bird and insect flower traits in Fritillaria L. (Liliaceae). Frotniers in Plant Sciences |  |
| F. carica Rix |  | Turkey | B | no | yes | no | no | 10.7 | 17.5 | 13.6 | 21.1 | [www.fritillariaicones.com](http://www.fritillariaicones.com/), Roguz et al. 2018. Evolution of bird and insect flower traits in Fritillaria L. (Liliaceae). Frotniers in Plant Sciences |  |
| F. caucasica (Boiss.) Grossh. |  | Caucasus, Iran, Turkey | B | no | yes | no | no | NA | 21.5 | 1.4 | 26.3 | [www.efloras.org](http://www.efloras.org/), [www.fritillariaicones.com](http://www.fritillariaicones.com/),Roguz et al. 2018. Evolution of bird and insect flower traits in Fritillaria L. (Liliaceae). Frotniers in Plant Sciences | BG,PK |
| F. chitralensis (auct.) B.Mathew |  | Pakistan, Afghanistan | B | yes | no | no | no | NA | 35 | NA | NA | www.efloras.org |  |
| F. cirrhosa D. Don |  |  | China | B | no | yes | no | yes | NA | 40 | NA | NA | [www.efloras.org](http://www.efloras.org/), [www.fritillariaicones.com](http://www.fritillariaicones.com/) |  |
| F. conica Boiss. |  | Greece | D | no | no | no | no | NA | 26 | NA | NA | [www.fritillariaicones.com](http://www.fritillariaicones.com/), Zhos and Siagou. 2017. A new yellow-flowered Fritillaria species (Liliaceae) from Mt. Tisseon, continental Greece and its taxonomic relationships. Phytotaxa |  |
| F. crassicaulis S.C.Chen |  |  | China | B | no | no | no | yes | NA | 45 | NA | NA | www.efloras.org |  |
| F. crassifolia Rix |  |  | Iran, Iraq, Turkey | B | no | no | no | yes | NA | 20 | NA | NA | [www.fritillariaicones.com](http://www.fritillariaicones.com/), Tekşen and Aytaç. 2011. The revision of the genus Fritillaria L. (Liliaceae) in the Mediterranean region (Turkey). Turkish Journal of Botany |  |
| F. dagana Turcz. |  |  | Siberia, Mongolia | B | no | yes | no | yes | NA | NA | NA | NA | www.efloras.org |  |
| F. dajinensis S.C.Chen |  | China | B | no | yes | no | yes | NA | 20.5 | NA | NA | www.efloras.org |  |
| F. davidii Franchet |  |  | China | B | no | no | no | yes | NA | 35 | NA | NA | [www.efloras.org](http://www.efloras.org/), [www.fritillariaicones.com](http://www.fritillariaicones.com/) |  |
| F. davisii Turrill |  | Greece | D | no | no | no | yes | 20 | 19 | NA | NA | [www.fritillariaicones.com](http://www.fritillariaicones.com/), Roguz et al. 2018. Evolution of bird and insect flower traits in Fritillaria L. (Liliaceae). Frotniers in Plant Sciences |  |
| F. delavayi Franchet |  |  | China | B | no | yes | no | yes | NA | 38.5 | NA | NA | www.efloras.org |  |
| F. drenovskii Degen and Stoj. |  |  | Greece | D | no | yes | no | yes | NA | NA | 35.8 | 29.1 | [www.fritillariaicones.com](http://www.fritillariaicones.com/), Roguz et al. 2018. Evolution of bird and insect flower traits in Fritillaria L. (Liliaceae). Frotniers in Plant Sciences |  |
| F. eastwoodiae R.M. Macfarlane. |  |  | North America | F | no | yes | no | yes | NA | 13.5 | NA | NA | www.efloras.org | CE |
| F. eduardii A. Regel ex Regel |  | Central Asia | B | yes | yes | no | no | 67.8 | 53.2 | 56.5 | 5.3 | [www.efloras.org](http://www.efloras.org/), Roguz et al. 2018. Evolution of bird and insect flower traits in Fritillaria L. (Liliaceae). Frotniers in Plant Sciences | BG |
| F. ehrhartii Boiss. & Orph. |  |  | Greece | D | no | no | no | no | NA | 19 | NA | NA | [www.fritillariaicones.com](http://www.fritillariaicones.com/), Zhos and Siagou. 2017. A new yellow-flowered Fritillaria species (Liliaceae) from Mt. Tisseon, continental Greece and its taxonomic relationships. Phytotaxa |  |
| F. elwesii Boiss. |  |  | Turkey | B | no | no | no | no | NA | 18 | NA | NA | Tekşen and Aytaç. 2011. The revision of the genus Fritillaria L. (Liliaceae) in the Mediterranean region (Turkey). Turkish Journal of Botany |  |
| F. falcata (Jeps.) Beetle |  |  | North America | F | yes | yes | yes | yes | NA | 18.5 | NA | NA | [www.efloras.org](http://www.efloras.org/), [www.fritillariaicones.com](http://www.fritillariaicones.com/) |  |
| F. ferganensis Losinsk |  |  | Kazahstan, Uzbekistan | B | no | yes | no | yes | NA | 30 | NA | NA | [www.efloras.org](http://www.efloras.org/) |  |
| F. fleischeriana Steud. and Hochst. ex Schult. and Schult.f. |  |  | Turkey | B | no | no | no | no | NA | NA | NA | NA | [www.fritillariaicones.com](http://www.fritillariaicones.com/), |  |
| F. forbesii Baker |  | Turkey | B | no | no | no | no | NA | 19.5 | NA | NA | [www.fritillariaicones.com](http://www.fritillariaicones.com/), Tekşen and Aytaç. 2011. The revision of the genus Fritillaria L. (Liliaceae) in the Mediterranean region (Turkey). Turkish Journal of Botany |  |
| F. frankiorum R.Wallis & R.B.Wallis |  | Turkey | B | no | yes | no | no | NA | NA | NA | NA | [www.fritillariaicones.com](http://www.fritillariaicones.com/) |  |
| F. gentneri Gilkey Madroño |  | North America | F | no | yes | no | yes | 9.3 | 28.6 | 54 | 31.1 | [www.efloras.org](http://www.efloras.org/), Roguz et al. 2018. Evolution of bird and insect flower traits in Fritillaria L. (Liliaceae). Frotniers in Plant Sciences | BG |
| F. gibbosa Boiss. |  | Caucasus, Iran, Afghanistan, Pakistan, Turkmenistan | B | yes | yes | yes | no | NA | 15.2 | NA | NA | [www.efloras.org](http://www.efloras.org/), [www.fritillariaicones.com](http://www.fritillariaicones.com/) | PK, LH |
| F. glauca Greene |  | North America | F | yes | no | no | yes | NA | 20 | NA | NA | www.efloras.org |  |
| F. graeca Boiss. and Sprun. |  |  | Greece | D | no | yes | no | yes | NA | 20 | 9.6 | 52.6 | [www.fritillariaicones.com](http://www.fritillariaicones.com/), Roguz et al. 2018. Evolution of bird and insect flower traits in Fritillaria L. (Liliaceae). Frotniers in Plant Sciences | BG |
| F. gussichiae (Degen and Dörfl.) Rix |  |  | Albania, Bulgaria, Greece, North Macedonia, Serbia | D | no | yes | no | no | 25.8 | 34.1 | 44.5 | 14.5 | Roguz et al. 2018. Evolution of bird and insect flower traits in Fritillaria L. (Liliaceae). Frotniers in Plant Sciences |  |
| F. hermonis Fenzl |  |  | Syria | B | no | yes | no | yes | NA | 23 | NA | NA | Rix. 2011. 711. Fritillaria Hermonis. Curtis’s Botanical Magazine, Tekşen and Aytaç. 2011. The revision of the genus Fritillaria L. (Liliaceae) in the Mediterranean region (Turkey). Turkish Journal of Botany |  |
| F. imperialis L. |  |  | Turkey, Syria, Lebanon | B | yes | yes | no | no | 34.8 | 35.7 | 205 | 13.6 | [www.efloras.org](http://www.efloras.org/), [www.fritillariaicones.com](http://www.fritillariaicones.com/) | BG |
| F. involucrata All. |  |  | France | D | no | yes | no | yes | NA | 32.5 | NA | NA | Rix. 2000. Plate 398. Fritillaria involucrata. Curtis's Botanical Magazine |  |
| F. japonica Miq. |  |  | Japan | C | no | yes | no | no | NA | NA | NA | NA | [www.fritillariaicones.com](http://www.fritillariaicones.com/) |  |
| F. kaiensis Naruh. |  | Japan | C | no | yes | no | yes | NA | NA | NA | NA | [www.fritillariaicones.com](http://www.fritillariaicones.com/) |  |
| F. karelinii (Fisch. ex D.Don) Baker |  | Central Asia | B | yes | yes | no | yes | NA | 12.5 | NA | NA | [www.efloras.org](http://www.efloras.org/), [www.fritillariaicones.com](http://www.fritillariaicones.com/) |  |
| F. koidzumiana Ohwi |  | Iran | B | no | yes | no | yes | 14 | 15 | NA | NA | Rix. 2005. 535. Fritillaria koidzumiana. The Board of Trustees of the Royal Botanic Gardens |  |
| F. kotschyana Herbert |  |  | Iran | B | no | yes | no | yes | NA | 40 | 55.3 | 24.3 | [www.efloras.org](http://www.efloras.org/), [www.fritillariaicones.com](http://www.fritillariaicones.com/),Roguz et al. 2018. Evolution of bird and insect flower traits in Fritillaria L. (Liliaceae). Frotniers in Plant Sciences | PK |
| F. kurdica Boiss. & Noë |  |  | Iran, Iraq, Turkey, Caucasus | B | no | no | no | yes | NA | 25 | NA | NA | [www.efloras.org](http://www.efloras.org/), [www.fritillariaicones.com](http://www.fritillariaicones.com/) | PK |
| F. latakiensis Rix |  |  | Turkey | B | no | yes | no | no | NA | 24 | NA | 37.6 | [www.fritillariaicones.com](http://www.fritillariaicones.com/), Roguz et al. 2018. Evolution of bird and insect flower traits in Fritillaria L. (Liliaceae). Frotniers in Plant Sciences | LH |
| F. latifolia Willd. |  | Turkey | B | no | yes | no | yes | NA | 35 | NA | NA | [www.efloras.org](http://www.efloras.org/), [www.fritillariaicones.com](http://www.fritillariaicones.com/) |  |
| F. liliacea Lindl. |  | North America | F | yes | yes | yes | no | NA | 13 | NA | NA | [www.efloras.org](http://www.efloras.org/), [www.fritillariaicones.com](http://www.fritillariaicones.com/) | CE |
| F. lusitanica Wikstr. |  |  | Spain, Portugal | D | no | yes | no | yes | NA | NA | NA | NA | [www.fritillariaicones.com](http://www.fritillariaicones.com/) |  |
| F. maximowiczii Freyn. |  |  | Russia, china | B | no | yes | no | yes | NA | 37.5 | NA | NA | [www.efloras.org](http://www.efloras.org/), [www.fritillariaicones.com](http://www.fritillariaicones.com/) |  |
| F. meleagris L. |  |  | Europe | D | no | yes | no | yes | NA | 10.5 | NA | NA | [www.efloras.org](http://www.efloras.org/), [www.fritillariaicones.com](http://www.fritillariaicones.com/) |  |
| F. meleagroides Patrin ex Schult.f |  | Eurasia | DB | no | yes | no | yes | NA | 27.5 | NA | NA | [www.efloras.org](http://www.efloras.org/), [www.fritillariaicones.com](http://www.fritillariaicones.com/) | PK |
| F. messanensis Rafin in Desv |  |  | Greece | D | no | yes | no | yes | NA | NA | NA | NA | [www.fritillariaicones.com](http://www.fritillariaicones.com/) | NATURAL POP. |
| F. michailovskyi Fomin |  |  | Turkey | B | no | yes | no | no | 18.9 | 23 | 3.6 | 17.4 | [www.efloras.org](http://www.efloras.org/), Roguz et al. 2018. Evolution of bird and insect flower traits in Fritillaria L. (Liliaceae). Frotniers in Plant Sciences | BG |
| F. micrantha A. A. Heller |  |  | North America | F | yes | no | yes | no | NA | 15 | NA | NA | [www.efloras.org](http://www.efloras.org/), [www.fritillariaicones.com](http://www.fritillariaicones.com/) |  |
| F. minima |  | Eastern Turkey | B | no | no | no | no | NA | 17 | NA | NA | Khaniki and Persson. 1997. Nectary morphology in South West Asian Fritillaria ( Liliaceae ). Nordic Journal of Botany | BG |
| F. minuta Rix. |  |  | Turkey | B | no | yes | no | no | 11.2 | 21.9 | 1.2 | 64.9 | Roguz et al. 2018. Evolution of bird and insect flower traits in Fritillaria L. (Liliaceae). Frotniers in Plant Sciences |  |
| F. monantha Migo |  |  | China | B | no | yes | no | yes | NA | 42.5 | NA | NA | [www.efloras.org](http://www.efloras.org/), [www.fritillariaicones.com](http://www.fritillariaicones.com/) |  |
| F. montana Hoppe ex W. D. J. Koch |  | Europe | D | no | yes | no | yes | NA | 22 | NA | NA | [www.efloras.org](http://www.efloras.org/), [www.fritillariaicones.com](http://www.fritillariaicones.com/) | BG, PK |
| F. muraiana Ohwi |  | Japan | C | no | yes | no | yes | NA | NA | NA | NA | [www.fritillariaicones.com](http://www.fritillariaicones.com/) |  |
| F. mutabilis Kamari |  |  | Greece | D | no | yes | no | yes | NA | NA | NA | NA | [www.fritillariaicones.com](http://www.fritillariaicones.com/) | BG |
| F. obliqua Ker Gawl. |  | Greece | D | no | yes | no | no | NA | 31 | NA | NA | [www.fritillariaicones.com](http://www.fritillariaicones.com/), Zhos and Siagou. 2017. A new yellow-flowered Fritillaria species (Liliaceae) from Mt. Tisseon, continental Greece and its taxonomic relationships. Phytotaxa |  |
| F. olgae Vved. |  |  | Central Asia | B | no | yes | no | yes | 19.4 | 24.8 | 75 | 30 | [www.efloras.or](http://www.efloras.org/)[g](http://www.efloras.org/), Roguz et al. 2018. Evolution of bird and insect flower traits in Fritillaria L. (Liliaceae). Frotniers in Plant Sciences |  |
| F. olivieri Baker |  |  | Iran | B | no | yes | no | yes | 20.6 | 33.8 | 11.6 | 35.7 | [www.efloras.org](http://www.efloras.org/), [www.fritillariaicones.com](http://www.fritillariaicones.com/), Roguz et al. 2018. Evolution of bird and insect flower traits in Fritillaria L. (Liliaceae). Frotniers in Plant Sciences |  |
| F. oranensis Pomel |  |  | North Africa | A | no | yes | no | no | NA | 30 | NA | NA | www.efloras.org |  |
| F. orientalis Adams |  |  | Turkey | B | no | yes | no | yes | NA | 30 | NA | NA | [www.efloras.org](http://www.efloras.org/), [www.fritillariaicones.com](http://www.fritillariaicones.com/) |  |
| F. pallidiflora Schrenk |  | Kazahstan, Uzbekistan | B | no | yes | no | yes | 32.7 | 38.3 | 26 | 43.7 | [www.efloras.org](http://www.efloras.org/), Roguz et al. 2018. Evolution of bird and insect flower traits in Fritillaria L. (Liliaceae). Frotniers in Plant Sciences |  |
| F. persica L |  |  | Middle East | B | yes | yes | yes | no | 19.7 | 19.3 | 4.3 | 47 | [www.fritillariaicones.com](http://www.fritillariaicones.com/), Roguz et al. 2018. Evolution of bird and insect flower traits in Fritillaria L. (Liliaceae). Frotniers in Plant Sciences | BG |
| F. phaeanthera Eastw. |  |  | North America | F | no | yes | no | no | NA | 12.5 | NA | NA | Macfarlane. 1978. On The Taxonomic Status Of Fritillaria Phaeanthera Eastw. (liliaceae). Madroño |  |
| F. pinardii Boiss. |  |  | Middle East | B | no | yes | no | no | NA | 17.5 | NA | NA | [www.fritillariaicones.com](http://www.fritillariaicones.com/), Tekşen and Aytaç. 2011. The revision of the genus Fritillaria L. (Liliaceae) in the Mediterranean region (Turkey). Turkish Journal of Botany |  |
| F. pluriflora Torrey ex Bentham |  |  | North America | F | yes | yes | yes | no | NA | 27.5 | NA | NA | [www.fritillariaicones.com](http://www.fritillariaicones.com/), www.efloras.org |  |
| F. pontica Wahlenb. |  |  | Albania, Bulgaria, Greece, Macedonia, Serbia | D | no | yes | no | yes | NA | NA | 5.6 | 16.1 | [www.fritillariaicones.com](http://www.fritillariaicones.com/), Roguz et al. 2018. Evolution of bird and insect flower traits in Fritillaria L. (Liliaceae). Frotniers in Plant Sciences | BG |
| F. przewalskii Maxim. |  | China | B | no | no | yes | no | NA | 25 | NA | NA | www.efloras.org |  |
| F. pudica (Pursh) Sprengel |  | North America | F | no | yes | no | yes | NA | 15 | NA | NA | [www.efloras.org](http://www.efloras.org/), [www.fritillariaicones.com](http://www.fritillariaicones.com/) |  |
| F. pyrenaica L. |  |  | France | D | no | no | no | no | NA | NA | 52.3 | 12.8 | [www.fritillariaicones.com](http://www.fritillariaicones.com/), Roguz et al. 2018. Evolution of bird and insect flower traits in Fritillaria L. (Liliaceae). Frotniers in Plant Sciences | BG |
| F. raddeana Regel. |  | Iran, Iraq, Turkey | B | yes | yes | no | yes | 44 | 33.3 | 8.7 | 50.1 | [www.fritillariaicones.com](http://www.fritillariaicones.com/), Roguz et al. 2018. Evolution of bird and insect flower traits in Fritillaria L. (Liliaceae). Frotniers in Plant Sciences | BG |
| F. recurva Benth. |  | North America | F | yes | yes | no | no | NA | 26 | 49.2 | 33.1 | [www.efloras.org](http://www.efloras.org/), Roguz et al. 2018. Evolution of bird and insect flower traits in Fritillaria L. (Liliaceae). Frotniers in Plant Sciences | BG |
| F. reuteri Boiss. |  |  | Turkey | B | no | yes | no | no | NA | 18 | NA | NA | [www.fritillariaicones.com](http://www.fritillariaicones.com/), Rix. 1974. Notes on Fritillaria (Liliaceae) in the Eastern Mediterranean Region, I &II. Kew Bulletin |  |
| F. rixii E. Zaharof |  | Greece | D | no | yes | no | yes | NA | 18.5 | NA | NA | [www.fritillariaicones.com](http://www.fritillariaicones.com/), Zaharof, E. 1986. Fritillaria rixii (Liliaceae), a new species from Euboea, Greece. Nordic Journal of Botany, |  |
| F. ruthenica Wikst. |  | Eurasia | DB | no | yes | no | no | 16.9 | 24.5 | NA | NA | [www.efloras.org](http://www.efloras.org/), [www.fritillariaicones.com](http://www.fritillariaicones.com/) |  |
| F. sewerzowii Regel. |  |  | Central Asia | B | yes | yes | yes | no | 9.3 | 29 | 23.7 | 61.9 | [www.fritillariaicones.com](http://www.fritillariaicones.com/), Roguz et al. 2018. Evolution of bird and insect flower traits in Fritillaria L. (Liliaceae). Frotniers in Plant Sciences |  |
| F. shikokiana Naruhashi |  | China | B | no | no | yes | no | NA | NA | NA | NA | [www.fritillariaicones.com](http://www.fritillariaicones.com/) | BG |
| F. sibthorpiana (Sm) Baker |  | Turkey | B | no | no | no | yes | 11.7 | 17.6 | NA | NA | [www.fritillariaicones.com](http://www.fritillariaicones.com/), Tekşen and Aytaç. 2011. The revision of the genus Fritillaria L. (Liliaceae) in the Mediterranean region (Turkey). Turkish Journal of Botany |  |
| F. sichuanica S.C.Chen |  |  | China | B | no | yes | no | yes | NA | 32.5 | NA | NA | www.efloras.org |  |
| F. sinica S.C.Chen |  | China | B | no | yes | no | yes | NA | 35 | NA | NA | [www.efloras.org](http://www.efloras.org/), [www.fritillariaicones.com](http://www.fritillariaicones.com/) |  |
| F. sororum Jim.Persson and K.Persson |  |  | Turkey | B | no | no | no | no | NA | 35 | NA | NA | [www.fritillariaicones.com](http://www.fritillariaicones.com/), Rix and Strange. 2014. 791. Fritillaria Sororum. Curtis’s Botanical Magazine |  |
| F. stenanthera (Regel) Regel |  | Central Asia | B | yes | yes | yes | yes | 15.5 | 18.4 | 0.6 | 46 | [www.fritillariaicones.com](http://www.fritillariaicones.com/), Roguz et al. 2018. Evolution of bird and insect flower traits in Fritillaria L. (Liliaceae). Frotniers in Plant Sciences | BG |
| F. straussii Bornm. |  | Turkey | B | no | no | no | no | NA | 18 | NA | NA | [www.fritillariaicones.com](http://www.fritillariaicones.com/), Rix. 1974. Notes on Fritillaria (Liliaceae) in the Eastern Mediterranean Region, I &II. Kew Bulletin |  |
| F. striata Ewastw. |  | North America | F | yes | yes | yes | no | NA | 27.5 | NA | NA | www.efloras.org |  |
| F. stribrnyi Velenovský |  |  | Eurasia | DB | no | yes | no | yes | NA | 24 | NA | NA | [www.fritillariaicones.com](http://www.fritillariaicones.com/), Notes on Fritillaria (Liliaceae) in the Eastern Mediterranean Region: IV |  |
| F. sulcisquamosa S.Y.Tang & S.C.Yueh |  |  | China | B | no | yes | no | yes | NA | NA | NA | NA | [www.fritillaria.org.uk](http://www.fritillaria.org.uk/) |  |
| F. taipaiensis P.Y.Li |  |  | China | B | no | yes | no | no | NA | 37.5 | NA | NA | www.efloras.org |  |
| F. theophrasti Kamari & Phitos |  |  | Greece | D | no | yes | no | yes | NA | NA | NA | NA | [www.fritillariaicones.com](http://www.fritillariaicones.com/) |  |
| F. thessala (Boiss.) Kamari |  |  | Greece | D | no | yes | no | yes | NA | NA | NA | NA | [www.fritillaria.org.uk](http://www.fritillaria.org.uk/) |  |
| F. thunbergii Miq. |  | Kazahstan, Uzbekistan | B | yes | yes | no | yes | NA | 30 | NA | NA | [www.fritillariaicones.com](http://www.fritillariaicones.com/), www.efloras.org | BG, PK |
| F. tokushimensis Akasawa, Katayama & T.Naito |  | Japan | C | no | yes | no | yes | NA | NA | NA | NA | [www.fritillariaicones.com](http://www.fritillariaicones.com/) |  |
| F. tortifolia X.Z.Duan & X.J.Zheng |  | China | B | no | no | no | no | NA | 30 | NA | NA | [www.fritillariaicones.com](http://www.fritillariaicones.com/), www.efloras.org |  |
| F. tubiformis Gren. and Godr |  | Europe | D | no | yes | no | no | NA | NA | NA | NA | [www.fritillaria.org.uk](http://www.fritillaria.org.uk/) | BG |
| F. unibracteata P.K.Hsiao & K.C.Hsia |  |  | China | B | no | yes | no | yes | NA | 26 | NA | NA | www.efloras.org |  |
| F. ussuriensis Maxim |  | Asia | B | no | yes | no | yes | 5.2 | 21.9 | NA | NA | [www.fritillariaicones.com](http://www.fritillariaicones.com/), Roguz et al. 2018. Evolution of bird and insect flower traits in Fritillaria L. (Liliaceae). Frotniers in Plant Sciences | BG |
| F. uva-vulpis RixF. verticillata Willd |  |  | Turkey | B | no | no | no | no | 13.4 | 20.8 | 12 | 47.8 | [www.fritillariaicones.com](http://www.fritillariaicones.com/), Roguz et al. 2018. Evolution of bird and insect flower traits in Fritillaria L. (Liliaceae). Frotniers in Plant Sciences | BG |
| F. verticillata Willd |  | Japan | C | yes | yes | no | no | 27.6 | 15.2 | NA | NA | [www.efloras.org](http://www.efloras.org/), [www.fritillariaicones.com](http://www.fritillariaicones.com/) |  |
| F. walujewii Regel |  |  | Kazahstan, Uzbekistan | B | no | yes | no | yes | NA | 40 | NA | NA | [www.efloras.org](http://www.efloras.org/), [www.fritillariaicones.com](http://www.fritillariaicones.com/) |  |
| F. whittallii Baker |  |  | Turkey | B | no | yes | no | yes | 24.3 | 30.7 | 13.5 | 50.9 | [www.efloras.org](http://www.efloras.org/), [www.fritillariaicones.com](http://www.fritillariaicones.com/), Roguz et al. 2018. Evolution of bird and insect flower traits in Fritillaria L. (Liliaceae). Frotniers in Plant Sciences | BG |
| F. yuminensis X.Z.Duanv |  | China | B | yes | yes | yes | no | NA | 17.5 | NA | NA | www.efloras.org |  |
| F. zagrica Stapf. |  | Iran | B | no | yes | no | no | NA | 15 | NA | NA | Tekşen and Aytaç. 2011. The revision of the genus Fritillaria L. (Liliaceae) in the Mediterranean region (Turkey). Turkish Journal of Botany |  |
